# Synthesis and evaluation of novel thiohydantoin derivatives for antidiabetic activity using in silico in vitro and in vivo methods

**DOI:** 10.1038/s41598-025-13538-7

**Published:** 2025-08-01

**Authors:** Asma Bukhari, Humaira Nadeem, Iqra Zulfiqar, Maira Anwar, Syed Muzzammil Masaud, Babar Murtaza

**Affiliations:** 1 Department of Pharmaceutical Chemistry, Riphah Institute of Pharmaceutical Sciences, Riphah International University, Street 41, 7th Ave, G-7/4, Islamabad, Pakistan; 2 Department of Basic Medical Sciences, Riphah Institute of Pharmaceutical Sciences, Riphah International University, Islamabad, Pakistan

**Keywords:** Thiohydantoin derivatives, Α-glucosidase inhibition, Α-amylase inhibition, molecular docking, *In vivo* antidiabetic evaluation, SAR analysis, Lipid profile, Drug discovery, Molecular medicine, Chemistry

## Abstract

Diabetes mellitus remains a global health challenge, necessitating the development of novel therapeutic agents. In this study, a series of thiohydantoin derivatives (FP1–FP7) were synthesized and evaluated for their in silico, in vitro, and in vivo antidiabetic potential. Molecular docking studies revealed strong binding affinities of derivatives towards α-glucosidase (PDB: 3wy1) and α-amylase (PDB: 3dhp), with FP4 exhibiting the most favorable interactions (− 7.8 kcal/mol with α-amylase and − 7.0 kcal/mol with α-glucosidase), involving hydrogen bonding and π-π stacking. During in vitro enzyme inhibition assay, FP4 demonstrated potent inhibitory activity against α-glucosidase and α-amylase, with IC₅₀ values of 129.40 and 128.90 µg/mL, respectively. The DPPH scavenging assay also indicated that FP4 had relatively strong antioxidant activity, with an IC₅₀ value of 39.7 µg/mL. In vivo antidiabetic efficacy was evaluated in STZ-induced diabetic rats over a period of 6-week. FP4 treated diabetic rats exhibited significantly reduced fasting blood glucose by 28.9% than in diabetic controls. In addition, HbA1C levels and diabetes-associated weight loss were significantly curtailed in these animals than untreated diabetic group. Further FP4 treated animals exhibited significantly decreased LDL and triglyceride levels and elevated HDL levels, suggesting a broader metabolic benefit. Taken together, our results suggest that thiohydantoin derivatives, particularly FP4, exhibited interesting antidiabetic and antihyperlipidemic activities warranting further pharmacokinetic and mechanistic investigations also potential for clinical translation and long term safety assessment.

**Supplementary Information:**

The online version contains supplementary material available at 10.1038/s41598-025-13538-7.

## Introduction

The hallmark of diabetes mellitus, a chronic metabolic disease, is persistent hyperglycemia brought on by compromised insulin secretion, insulin action, or both. Among the therapeutic strategies, one effective approach to managing postprandial hyperglycemia is the inhibition of carbohydrate-hydrolyzing enzymes, particularly α-glucosidase and α-amylase, in the digestive system. These enzymes are responsible for breaking down dietary carbohydrates into absorbable monosaccharides. By inhibiting these enzymes, the absorption of glucose is delayed, which is a critical therapy for the management of hyperglycemia in type 2 diabetes and borderline diabetic patients^1,2^.

The discovery of α-glucosidase and α-amylase inhibitors has gained significant attention due to their potential to manage hyperglycemia with minimal side effects. Dietary inhibitors, such as voglibose, acarbose, and miglitol, are widely used in clinical practice and act by competitively inhibiting α-glucosidase in the brush border of the small intestine. Consequently, they delay the hydrolysis of carbohydrates, reducing postprandial hyperglycemia^3^. However, the long-term use of these inhibitors is associated with adverse effects, including diarrhea, flatulence, abdominal discomfort, and hepatotoxicity^4^. These limitations have prompted extensive research into the development of safer, more effective inhibitors with additional therapeutic benefits, such as targeting obesity and even viral or cancer diseases^5^.

Hydantoins, five-membered heterocyclic compounds with a cyclic urea core, are versatile pharmacophores with broad therapeutic applications. Originally synthesized by the Baeyer group via the hydrogenation of allantoin, hydantoins have since been recognized for their diverse pharmacological profiles. This class of compounds provides multiple functionalization opportunities due to their small size, four derivatizable positions, and hydrogen donor/acceptor properties. Clinically approved hydantoin derivatives include anticonvulsants such as phenytoin, mephenytoin, ethotoin, and fosphenytoin, muscle relaxants like nitrofurantoin and dantrium, and androgen receptor antagonists such as nilutamide and enzalutamide^6,7^. These compounds illustrate the structural adaptability and therapeutic potential of the hydantoin scaffold.

Thiohydantoins, sulfur analogs of hydantoins, exhibit similar versatility in their pharmacological activities. The sulfur atom can occupy different positions within the heterocyclic ring, leading to various derivatives such as 2-thiohydantoins, 4-thiohydantoins, and 2, 4-dithiohydantoins^8^. Among these, 2-thiohydantoins have gained significant attention due to their unique chemical properties and wide-ranging biological activities. These compounds serve as intermediates in peptide synthesis and are utilized in diverse applications, including fungicides, herbicides, catalysts, and textile printing^9^. Moreover, 2-thiohydantoins have demonstrated remarkable therapeutic potential, exhibiting antimicrobial^10^, antibacterial^11^, anticancer^12,13^, and anti-inflammatory activities^8^.

The inhibition of α-glucosidase and α-amylase has emerged as a key strategy for reducing postprandial hyperglycemia, thereby managing diabetes and mitigating its associated complications. By delaying carbohydrate digestion and glucose absorption, these inhibitors play a pivotal role in controlling plasma glucose levels. However, there remains a pressing need for safer, more potent inhibitors with minimal side effects. Thiohydantoins, with their reactive cyclic thiourea core and multi-functional heterocyclic scaffold, have shown promise in addressing this need. Their chemical structure allows for straightforward modifications, resulting in derivatives with enhanced biological activity^14^.

Notably, thiohydantoin derivatives have demonstrated potent inhibitory activity against key therapeutic targets such as NADPH oxidase (NOX)^15^, human CB1 cannabinoid receptors^16,17^, and enzymes involved in microbial and cancer pathogenesis. Their hypolipidemic, antitubercular, and antimutagenic properties further highlight their pharmacological versatility^18^. Given their broad-spectrum biological activities and minimal structural modifications required to enhance potency, 2-thiohydantoins represent a promising class of compounds for antidiabetic drug development.

The present study focuses on the synthesis of novel thiohydantoin derivatives (FP1–FP7) designed to target **α-glucosidase**, a key enzyme involved in carbohydrate metabolism and a validated target for antidiabetic therapy^19,20^. These derivatives integrate the **thiohydantoin core**—a privileged heterocyclic scaffold recognized for its diverse biological activities including antidiabetic, antimicrobial, anticancer, and anti-inflammatory properties^21,22^ with strategically substituted aromatic rings and functional groups such as **methoxy**,** chloro**,** methylamino**, and **carbonyl moieties**. The **thioxo group** in the hydantoin ring enhances lipophilicity and facilitates stronger **hydrophobic interactions** with the enzyme active site^23,24^. Meanwhile, **aromatic substituents** support π–π stacking and van der Waals contacts, contributing to improved **binding affinity** and **selectivity** toward α-glucosidase^25^.

Furthermore, **electron-donating** and **electron-withdrawing groups** (e.g., –OMe, –Cl) were incorporated to modulate the electronic density of the aromatic ring, fine-tuning **enzyme interaction energies** and **selectivity profiles**^26^. **Amine functionalities**, including secondary and methylamino groups, serve as **hydrogen bond donors or acceptors**, enhancing molecular recognition within the enzyme’s active pocket^27^. This rational combination of functionalities was specifically designed to improve not only **enzyme inhibition** but also **pharmacokinetic traits** such as **solubility**, **lipophilicity**, and **metabolic stability**^28^. Compared to earlier reported thiohydantoin-based α-glucosidase inhibitors^29^, the present derivatives offer a **novel substitution pattern** and **optimized functional diversity**, thereby expanding the chemical space for potential **multi-target antidiabetic agents**.This investigation not only expands the scope of thiohydantoin-based therapeutics but also contributes to the ongoing efforts to develop effective antidiabetic agents with minimal side effects.

## Materials and methods

### Chemicals and equipment

Daejung (South Korea) and Alfa-Aesar (Germany) provided all of the raw ingredients. Digital Sanyo-Gallenkamp equipment was used to record the final goods’ uncorrected melting points. The Bruker AM-300 was used to record the ^1^HNMR spectra in DMSO-d6 (300–400 MHz) and CDCl_3_ while the ^13^C NMR spectra were recorded at 75 MHz. An FTIR spectrophotometer (Alpha Bruker-ATR eco ZnSe, vmax in cm-1) was used to record the FTIR spectra. Thin layer chromatography (TLC) was used to track each reaction’s development. P-toulenesulphonyl chloride, amino acid, sodium carbonate, pyridine, acetic anhydride, ammonium thiocynate, acetanilide, benzoic acid, chloro sulfonic acid, 4-chloro benzoic acid, 2-methoxy benzoic acid, toluic acid, acetophenone, distilled water, ethyl alcohol, methyl alcohol and other reagents were used for the synthesis.

### Experimental animals

The adult male Sprague Dawley rats utilized in this investigation were obtained from Riphah International University’s (RIPS-RIU) domestic facility. The obtained animals were 10–12 weeks old and weighed 250–290 g. Controlled circumstances such as a light/dark cycle, 45–55% humidity, and a temperature of 22 ± 2 ◦C were provided by this animal house facility. Every animal had unlimited access to food and water. According to the Institute of Laboratory Animal Resources, Commission on Life Sciences University, National Research Council (1996) regulation, all standard requirements were adhered to, as authorized by the REC (research and ethical committee) RIU, authorization number REC/RIPS/2022/29.

All animals were humanely euthanized under deep anesthesia at the end of the experimental period. Anesthesia was induced using intraperitoneal injection of ketamine (100 mg/kg) and xylazine (10 mg/kg). Once deep anesthesia was confirmed, euthanasia was carried out via cervical dislocation, following the institutional and international ethical guidelines for the care and use of laboratory animals.

### General procedure for the synthesis of 2-Thiohydantoin derivatives

**Scheme 1:**
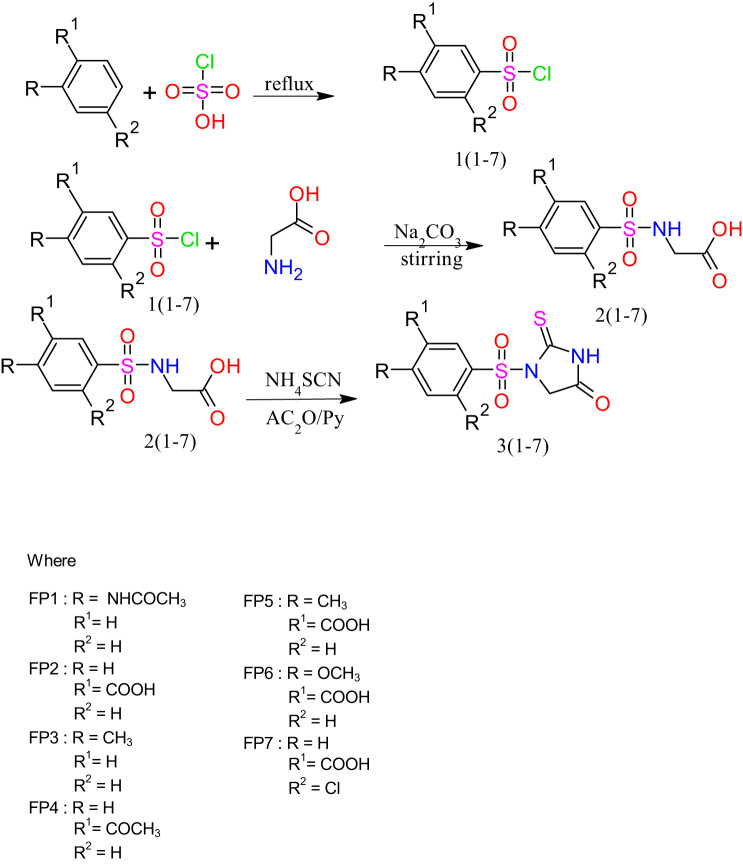
General procedure of synthesis of Thiohydantoin derivatives.

2-Thiohydantoin derivatives (FP1-FP7) were synthesized based on three-step reaction as according to the given scheme 1. Primarily, chlorosulfonic acid (ClSO_3_H) was reacted with different compound to yield sulfonyl derivatives. In the second step sulfonamide was generated through the reaction of amino acids and sulfonyl derivatives. Finally, 2-thiohydantoin derivatives FP1-FP7 were synthesized from the reaction of sulfonamides with ammonium thiocynate (NH_4_SCN) in the presence of acetic anhydride (C_4_H_6_O_3_) and pyridine (C_5_H_5_N) at 90 °C.

Substituted benzene derivatives were dissolved in concentrated chlorosulfonic acid (5 equivalent) with stirring for 30 min at room temperature. The mixture was refluxed for 2 h at 60-70 °C until the completion of reaction. The reaction progress was monitored through TLC. Upon completion (confirmed via TLC), the reaction mixture was allowed to cool gradually to ambient temperature and was then rapidly poured onto an excess of crushed ice under continuous stirring. The precipitated sulfonyl derivative was immediately filtered, washed with cold water, and dried under vacuum at room temperature to prevent hydrolysis of sulfonyl chloride functionalities. The crude products were used directly in the next step or recrystallized if needed^30,31^. as shown in Scheme 1.

A mixture of amino acids (12.5 mmol) in water (15 ml) and sodium carbonate (15 mmol) was stirred continuously until clear solution is obtained. After cooling the mixture to −5 °C, respective benzenesulphonyl chloride (15 mmol) was added over a period of one hour. For 48 h, the slurry was continuously swirled at room temperature. The reaction progress was monitored through TLC. After the completion, 20% aqueous hydrochloric acid was added to bring its pH 2. Solid separated was filtered and washed with water and dried^32^.

A mixture of 2 mmol of sulfonamide, 2.4 mmol of NH_4_SCN, and 4 mmol of acetic anhydride in anhydrous pyridine (1 ml) was heated to 90 °C for an hour. Then it was cooled to room temperature and 50 ml of cold water was added to the solution and it stirred for one hour at room temperature. After filtering out the precipitates and washing with water to get rid of extra pyridine, the precipitates was recrystallized from EtOH: H_2_0 (1:1)^33^.

The progress of each reaction step (sulfonylation, sulfonamide formation, and final thiohydantoin synthesis) was monitored using thin-layer chromatography (TLC) on pre-coated silica gel 60 F₂₅₄ plates (Merck, 0.25 mm thickness). For sulfonyl derivatives, an eluent system of n-hexane : ethyl acetate (7:3) was used. For sulfonamides and thiohydantoin derivatives (FP1–FP7), a mobile phase of chloroform : methanol (9:1) was employed. TLC spots were visualized under UV light at 254 and 365 nm, and by exposure to iodine vapor where applicable.

#### *N-[4-(4-oxo-2-sulfanylideneimidazolidine-1-sulfonyl)phenyl]acetamide 3(FP1)*

C_11_H_11_N_3_O_4_S_2_; White powder; Yield 70%; m.p 252–255 °C; FT-IR (Neat, ν cm⁻¹): 3572 (N–H), 3443 (C-H), 1756 (C = O), 1619 (amide stretching, N − C = O),1242 (C = C, aromatic),1077 (C = S); ¹H NMR (400 MHz, CDCl_3_,, δ = ppm): 2.4 (CH₃, s), 4.6.(CH_2,_ s), 7.4-8.0 (Ar-H, m), 8.3 (NH, s); ¹³C NMR (100 MHz, CDCl_3_, δ = ppm): 21.8 (CH₃), 51.8 (CH_2_), 126.63 (Aromatic C), 127.8 (Aromatic C), 127.8 (Aromatic C), 128.7 (Aromatic C),128.8 (Aromatic C), 129.3 (Aromatic C), 165.8 (C = O), 168.10 (C = O), 171.0 (C = S); Anal. Calcd for C_11_H_11_N_3_O_4_S_2_: C (42.16%), H (3.54%), N (13.41%); Found: C (42.10%), H (3.50%), N (13.38%); MS-EIMS (m/z): 314.0 [M + H]⁺, 249.1 (100), 270.1 (25) 207.0 (60).

#### *3-(4-oxo-2-sulfanylideneimidazolidine-1-sulfonyl)benzoic acid* 3(FP2)

C_10_H_8_N_2_O_5_S_2_; White powder; Yield 65%; m.p 226–228 °C; FT-IR (Neat, ν cm⁻¹):3817 cm⁻¹ (O-H) 3644 (N–H), 2920 (C-H), 1711 (C = O), 1574 (amide stretching, N − C = O), 1256 (C = C, aromatic) 1084 (C = S);¹H NMR (400 MHz, CDCl_3_, δ = ppm): 4.62(CH_2,_ s), 7.4-8.0 (Ar-H, m), 8.3 (NH, s);¹³C NMR (100 MHz, CDCl_3_, δ = ppm): 54.0 (CH_2_),127.0 (Aromatic C), 127.8 (Aromatic C), 127.8 (Aromatic C), 128.1 (Aromatic C), 129.1(Aromatic C), 129.4 (Aromatic C), 164.2 (C = O), 180.1 (C = S); Anal. Calcd for C_10_H_8_N_2_O_5_S_2_: C (39.99%), H (2.68%), N (9.33%);Found: C (39.94%), H (2.66%), N (9.31%);MS-EIMS (m/z): 301.3 [M + H]⁺, 240.1(100), 285.5 (10), 91.1 (92),

#### *1-(4-methylbenzene-1-sulfonyl)−2-sulfanylideneimidazolidin-4-one* 3(FP3)

C_10_H_10_N_2_O_3_S_2_; White powder; Yield 75%; m.p 270–272 °C; FT-IR (Neat, ν cm⁻¹): 3092 (N–H), 3047 (C-H), 1675 (C = O), 1586 (amide stretching, N − C = O), 1278 (C = C, aromatic) 1126 (C = S); ¹H NMR (400 MHz, CDCl_3_, δ = ppm): 2.5 (CH₃, s),4.89 (CH_2,_ s), 7.2–7.7 (Ar-H, m), 7.7 (NH, s); ¹³C NMR (100 MHz, CDCl_3_, δ = ppm): 21.1 (CH₃), 54.0 (CH_2_), 127.0 (Aromatic C), 127.8 (Aromatic C), 127.8 (Aromatic C), 128.1(Aromatic C), 129.1(Aromatic C), 129.4 (Aromatic C), 164.2 (C = O), 180.1 (C = S); Anal. Calcd for C_10_H_10_N_2_O_3_S_2_: C (44.44%), H (3.70%), N (10.37%); Found: C (44.41%), H (3.68%), N (10.36%);MS-EIMS (m/z): 271.5 ([M + H]⁺, 100%), 256.0 (10), 209.1 (56), 179.9 (100),

#### *1-(3-acetylbenzene-1-sulfonyl)−2-sulfanylideneimidazolidin-4-one* 3(FP4)

C_11_H_10_N_2_O_4_S_2_;White powder; Yield 70%; m.p 212–215 °C; FT-IR (Neat, ν cm⁻¹): 3783 (N–H), 2257 (C-H), 1726 (C = O), 1667 (amide stretching, N − C = O), 1398 (C = C, aromatic) 1022 (C = S);¹H NMR (400 MHz, CDCl_3_, δ = ppm): 2.5 (CH₃, s), 4.61(CH_2,_ s), 6.9-8.0 (Ar-H, m), 9.87 (NH, s);¹³C NMR (100 MHz, CDCl_3_, δ = ppm): 22.0 (CH₃), 53.8 (CH_2_), 126.6 (Aromatic C), 127.8 (Aromatic C), 127.8 (Aromatic C), 128.1 (Aromatic C), 129.0 (Aromatic C), 134.3 (Aromatic C), 165.7 (C = O), 168.1 (C = O), 179.0(C = S); Anal. Calcd for C_11_H_10_N_2_O_4_S_2_: C (44.30%), H (3.38%), N (9.39%);Found: C (44.28%), H (3.35%), N (9.36%); MS-EIMS (m/z): 299.2 ([M + H]⁺, 100%), 270.3 (24), 198.2 (15), 109.2 (95).

#### *2-methyl-5-(4-oxo-2-sulfanylideneimidazolidine-1-sulfonyl)benzoic acid* 3(FP5)

C_11_H_10_N_2_O_5_S_2_; White powder; Yield 75%; m.p 221–224 °C; FT-IR (Neat, ν cm⁻¹):3290 cm⁻¹ (O-H) 3355 (N-H), 2972 (C-H), 1728 (C = O), 1615 (amide stretching, N − C = O), 1295 (C = C, aromatic) 1200(C = S); ¹H NMR (400 MHz, DMSO-d6, δ = ppm): 2.4 (CH₃, s),4.79(CH_2,_ s) 7.4–7.9 (Ar-H, m), 12.6 (NH, s); ¹³C NMR (100 MHz, CDCl_3_, δ = ppm): 22.0 (CH₃), 54.2 (CH_2_), 127.0 (Aromatic C), 127.8 (Aromatic C), 127.8 (Aromatic C), 128.1(Aromatic C), 129.1(Aromatic C), 129.4 (Aromatic C),,164.2 (C = O), 168.2 (C = O), 178.2 (C = S); Anal. Calcd for C_11_H_10_N_2_O_5_S_2_: C (42.03%), H (3.18%), N (8.91%); Found: C (42.00%), H (3.16%), N (8.88%); MS-EIMS (m/z): 315.2 ([M + H]⁺, 100%), 270.2 (21), 109.1 (100),

#### *2-methoxy-5-(4-oxo-2-sulfanylideneimidazolidine-1-sulfonyl)benzoic acid* 3(FP6)

C_11_H_10_N_2_O_6_S_2_; White powder; Yield 70%; m.p 230–232 °C; FT-IR (Neat, ν cm⁻¹): 3367 (N–H), 3290 cm⁻¹ (O-H), 2978 (C-H), 1728 (C = O), 1620 (amide stretching, N − C = O), 1300 (C = C, aromatic) 1160 (C = S);¹H NMR (400 MHz, DMSO-d6, δ = ppm): 3.8 (OCH₃, s),4.10(CH_2,_ s) 6.5–7.9 (Ar-H, m), 8.2 (NH, s); ¹³C NMR (100 MHz, CDCl_3_, δ = ppm): 54.0 (CH_2_), 56.1 (OCH₃), 127.0 (Aromatic C), 127.8 (Aromatic C), 127.8 (Aromatic C), 128.1(Aromatic C), 129.1(Aromatic C), 129.4 (Aromatic C), 162.2 (C = O), 164.6(C = O) 170.1 (C = S); Anal. Calcd for C_11_H_10_N_2_O_6_S_2_: C (39.99%), H (3.05%), N (8.48%); Found: C (39.96%), H (3.03%), N (8.46%);MS-EIMS (m/z): 331.2 ([M + H]⁺, 100%), 270.2 (25), 109.1(100).

#### *4-chloro-3-(4-oxo-2-sulfanylideneimidazolidine-1-sulfonyl)benzoic acid* 3(FP7)

C_10_H_7_ClN_2_O_5_S_2_;Light brown powder; Yield 80%; m.p 243–245 °C; FT-IR (Neat, ν cm⁻¹): 3365 (N–H), 3290 cm⁻¹ (O-H), 2978 (C-H), 1729 (C = O), 1618 (amide stretching, N − C = O), 1295 (C = C, aromatic) 1080 (C = S);¹H NMR (400 MHz, CDCl_3_, δ = ppm):4.00 (CH_2,_ s), 6.5–7.5 (Ar-H, m), 8.2 (NH, s);¹³C NMR (100 MHz, DMSO-d6, δ = ppm): 52.0 (CH_2_), 127.0 (Aromatic C), 127.8 (Aromatic C), 127.8 (Aromatic C), 128.1(Aromatic C), 129.1(Aromatic C), 129.4 (Aromatic C), 162.2 (C = O), 164.6(C = O),176.1 (C = S) Anal. Calcd for C_10_H_7_ClN_2_O_5_S_2_: C (35.89%), H (2.11%), Cl (10.60%), N (8.37%);Found: C (35.85%), H (2.08%), Cl (10.57%), N (8.34%);MS-EIMS (m/z): 335.2 ([M + H]⁺, 100%), 300.4 (20), 198.2 (15),109.2 (97).

## Pharmacological evaluation

### In vitro assays

#### Antioxidant activity

##### Assessment of free radical DPPH scavenging activity

The antioxidant activity of synthesized compounds was evaluated using the DPPH free-radical scavenging assay, following the methodology outlined by^34^. This assay is based on the principle that the stable free radical DPPH undergoes a color change from deep purple to yellow upon reduction, indicating hydrogen donation from antioxidant compounds. Different concentrations (3.12, 6.25, 12.5, 25, 50, 100, and 200 µg/mL) of test compounds were prepared in methanol. A 200 µL aliquot of each concentration was mixed with 50 µL of freshly prepared DPPH solution (0.659 mM). The reaction mixtures were incubated at room temperature for 20 min in the dark. Absorbance was recorded at 510 nm using an ELISA microplate reader. Trolox was employed as the reference antioxidant, and the percentage of inhibition was calculated using the following equation:

% Inhibition = [(A0 − A1)/A0] × 100.

Where **A0** is the absorbance of the control, and **A1** is the absorbance of the sample^34^.

## α-Glucosidase inhibitory activity assay

The ability of the synthesized compounds to inhibit α-glucosidase was determined as per the method described by^35^. The enzyme α-glucosidase (EC3.2.1.20, from *Saccharomyces cerevisiae*), the standard inhibitor acarbose, and the substrate *p*-nitrophenyl-β-D-glucopyranoside were procured from Sigma-Aldrich. The enzyme was dissolved in 50 mM potassium phosphate buffer (pH 6.8), while test compounds and acarbose were prepared in DMSO (final concentration: 10%). Each well of a 96-well plate contained 20 µL of synthesized compound/acarbose at different concentrations (1, 10, 100, and 1000 µg/mL), 20 µL of enzyme solution, and 135 µL of buffer. After incubation at 37 °C for 10 min, the reaction was initiated by adding 25 µL of substrate (4 mM) and further incubating at 37 °C for 20 min. The absorbance was measured at 405 nm. Acarbose was used as the reference inhibitor, and the IC_50_ values were calculated using GraphPad Prism software version 9.5.1 for Windows (GraphPad Software, San Diego, California, USA; https://www.graphpad.com/^36^.

### α-Amylase inhibitory activity assay

The inhibitory potential of the synthesized derivatives against α-amylase was determined using the DNSA (3,5-dinitrosalicylic acid) colorimetric method, as reported by Bernfeld^37^. The α-amylase enzyme (from Sigma-Aldrich) was prepared in 20 mM sodium phosphate buffer (pH 6.9). The synthesized compounds and acarbose were dissolved in DMSO (10% final concentration). A reaction mixture containing 100 µL of test compound/acarbose (0.1, 1, 10, and 100 µg/mL) and 100 µL of α-amylase solution was incubated at 25 °C for 30 min. After incubation, 100 µL of DNSA reagent was added, and the mixture was heated at 85 °C for 15 min. The reaction was stopped by cooling, and the absorbance was measured at 595 nm. A blank control was prepared to adjust background absorbance, and the percentage inhibition was calculated^37^.

### Cytotoxicity assessment using MTT assay

The cytotoxic effects of the selected compounds were evaluated against the 3T3 fibroblast cell line (Sigma-Aldrich, product no. 90020107) using the MTT (3-(4,5-dimethylthiazol-2-yl)−2,5-diphenyl tetrazolium bromide) assay, following the protocol established by Mosmann^38^. A cell suspension containing 1 × 10⁵ cells per well was seeded into a 96-well plate and incubated at 37 °C with 5% CO₂ for 48 h. The cells were then treated with different concentrations of test compounds (4, 16, 63, 250, and 1000 µg/mL, dissolved in DMSO) and incubated for an additional 48 h. Following incubation, 180 µL of MTT solution (5 mg/mL in PBS) was added to each well and incubated for 4 h. The formazan crystals formed were solubilized, and absorbance was measured at 570 nm using an ELISA reader. Celecoxib was used as a positive control^38^.

### In vivo pharmacological evaluation

#### Induction of diabetes

Diabetes will be induced in rats (*n* = 6 per group) by a single intraperitoneal injection of 60-100 mg/kg body weight (BW) streptozotocin dissolved in normal saline. After 5 days of streptozotocin injection, the tail vein blood will be collected to determine the fasting blood glucose (FBG) level. Only rats with FBG over 200 mg/dl will be considered diabetic and used for further experimentation^39^.

#### Oral glucose tolerance test (OGTT) assay

This assay will be performed in animals after fasting for an overnight (12 h). Then, rats will receive a bolus of glucose (2.0 g/kg body weight) followed by test molecule or control and plasma glucose will be measured from blood collected from tail vein at 0, 30, 60, 90 and 120 min after glucose administration. Plasma glucose levels will be analyzed using commercially available glucometer kits^40^.

#### Effect of synthetic agents on fasting blood glucose (FBG), body weight (BW) and HbA1C

Diabetic Rats (*n* = 6 per group) will be randomly assigned to groups of six animals each in order to examine the impact of synthetic derivatives on FBG level, BW, and HbA1C. The course of treatment will last for six weeks. At the beginning of the experiment and at weekly intervals during the first 42 days of therapy, the FBG level and BW will be measured. Rats will be denied food for 12 h while fasting, but they will have unrestricted access to water. Commercial glucose oxidase kits will be used to measure the blood glucose level. Blood 7 will be collected through cardiac puncture at the end of study period and levels of HbA1C will be determined. Metformin will be used as standard for comparison purpose.

#### Determination of physiological parameters

Animals will be sacrificed after 7 weeks of treatment and parameters including LDL, triglycerides, HDL cholesterol, and glucose will be determined by using KONELAB SYSTEM (Thermo Fisher Scientific, Finland) according to manufacturer’s guidelines.

#### In silico drug design

The 3D structures of the synthesized sulfonyl derivatives (compounds 1–7) will be drawn using ChemBioDraw Ultra 14.0. These structures will be saved in MDL SDF format and subsequently converted to PDB format. Ligand preparation, including energy minimization and conversion to PDBQT format, will be performed using PyRx 0.8 (which integrates Open Babel for file format conversion and structure optimization).The crystal structures of α-amylase and α-glucosidase will be retrieved from the Protein Data Bank (PDB). Proteins will be visualized, prepared (removal of water molecules and ligands), and analyzed using PyMOL 2.5.0. AutoDock Tools (AutoDock 4.2.6) will be employed for grid box generation and docking calculations. To validate the docking protocol, re-docking of the native ligand into the active site of the protein will be performed. The accuracy of docking will be evaluated by calculating the Root Mean Square Deviation (RMSD) between the docked pose and the crystallographic pose. The docking results will be clustered according to the root mean 8 square deviations (RMSD) of 2.0 Å. The structures with the relative lower binding free energy and the most cluster members will be chosen, for the best docking conformation.

The selection of α-glucosidase (PDB ID: 3WY1) and α-amylase (PDB ID: 3DHP) for molecular docking was based on their high-resolution crystal structures, well-characterized active sites, and established relevance in inhibitor screening studies. PDB ID 3WY1, representing Saccharomyces cerevisiae α-glucosidase (1.80 Å), is frequently employed in computational studies due to its conserved catalytic residues (Asp214, Glu276, Asp349) and structural similarity to human intestinal α-glucosidase^41,42^. Likewise, PDB ID 3DHP, derived from Sus scrofa pancreatic α-amylase (1.90 Å), shares over 85% sequence identity with the human enzyme and provides a reliable model for studying interactions with active site residues Asp197, Glu233, and Asp300^43,44^. Both structures have been widely utilized in virtual screening and SAR studies^45,46^, making them suitable templates for evaluating the binding affinity and interaction profiles of the synthesized thiohydantoin derivatives.

#### In silico ADMET prediction

The in silico ADMET profiling of FP1–FP7 was conducted using the PKCSM online tool to predict pharmacokinetic and toxicity parameters^28^. Key properties, including absorption (intestinal permeability, Caco-2 permeability), distribution (BBB penetration, plasma protein binding), metabolism (CYP enzyme interactions), excretion (clearance, renal involvement), and toxicity (hERG inhibition, hepatotoxicity), were analyzed. The findings provided crucial insights into the drug-like behavior and safety profile of the synthesized compounds. This computational approach facilitated early-stage evaluation, aiding in the selection of promising candidates for further biological investigations.

##### Statistical analysis

Statistical analysis of data will be done by using student’s t-test and one way analysis of variance (ANOVA) followed by appropriate post hoc test. All the values of data will be expressed as means (± S.E.M). The *P* < 0.05 will be considered as significant for analysis of data^47^.

## Results and discussion

### Synthesis and characterization

The synthesis was performed following **scheme-1**.The reaction proceeds via electrophilic aromatic substitution, where the electrophilic sulfur trioxide (SO₃H⁺) species, generated in situ from chlorosulfonic acid, attacks the electron-rich aromatic ring of the substrate, leading to the formation of aryl sulfonic acid derivatives 1(1–7) after proton elimination and stabilization of the sulfonyl group on the aromatic system.The successful synthesis of FP1–FP7 was confirmed through a combination of FTIR, ¹H NMR, ¹³C NMR, mass spectrometry, and elemental analysis. The FTIR spectra provided critical evidence of key functional groups present in the synthesized thiohydantoin derivatives. The characteristic stretching vibrations of the N–H group were observed in the range of 3360–3370 cm⁻¹, indicating the presence of the amide functionality. Strong absorption bands between 1726 and 1742 cm⁻¹ confirmed the presence of the carbonyl (C = O) functional group, essential for the thiohydantoin core structure. The presence of sulfonyl (-SO₂) groups in selected derivatives was verified by strong peaks appearing between 1290 and 1300 cm⁻¹, while C–N stretching bands were observed around 1100–1150 cm⁻¹, confirming the successful incorporation of nitrogen-linked substituents. These spectral findings affirmed the expected chemical functionalities in the synthesized compounds. **(Supplementary data Figure ****S1****-S5)**

¹H NMR spectra further confirmed the molecular structures of FP1–FP7 by identifying proton environments specific to the synthesized derivatives. The spectra exhibited multiple signals in the aromatic region between δ 6.4–7.9 ppm, corresponding to the expected aromatic protons. The NH proton signal was observed as a singlet at δ 8.1–8.4 ppm, validating the presence of the thiohydantoin core. For derivatives containing additional substituents, characteristic signals further substantiated their structures. In FP3–FP6, the presence of methyl (-CH₃) and methoxy (-OCH₃) groups was confirmed by distinct singlets at δ 2.1–2.4 ppm and δ 3.8 ppm, respectively. **(Supplementary data Figure S6-S11)**

Although singlets around δ 8.1–8.4 ppm were assigned to NH protons in the ¹H NMR spectra of FP1–FP6, D₂O exchange experiments were not conducted to confirm their exchangeability, which is a limitation of the current spectral confirmation, largely due to budget constraints.

The ¹³C NMR spectra provided further validation, revealing distinct carbonyl carbon signals between δ 160–180 ppm, indicative of the thiohydantoin framework. Aromatic carbon peaks were observed in the range of δ 115–145 ppm, while substituted alkyl carbons, such as methyl and methoxy groups, appeared at δ 10–60 ppm.**(Supplementary data Figure S12**,** Figure S13 and Figure S14)**.

Mass spectrometric analysis of FP1–FP7 confirmed the molecular integrity of the synthesized compounds. The molecular ion peaks ([M + H]⁺) in the spectra corresponded well with the calculated molecular weights of each derivative, providing strong evidence for successful synthesis. Fragmentation patterns were consistent with the expected breakdown pathways of the thiohydantoin core, displaying stable fragment ions that further validated the chemical structure. The stability of these peaks and the absence of unexpected mass losses indicated the purity and correct composition of the synthesized molecules. **(Supplementary data Figure S15 to Figure S17)**

Elemental analysis provided additional confirmation by comparing experimentally obtained percentages of carbon, hydrogen, nitrogen, oxygen, and sulfur with their theoretical values. The obtained data exhibited close agreement with the calculated compositions, with deviations within acceptable limits, confirming the high purity and successful formation of FP1–FP7. Collectively, these analytical techniques conclusively validated the successful synthesis of the targeted thiohydantoin derivatives, ensuring their structural authenticity and suitability for further biological and pharmacological evaluation.

### Pharmacological evaluation

#### In vitro analysis

##### Antioxidant potential

**Table 1:** DPPH free radical scavenging activity of FP1–FP7.

Concentration (µg/mL)	FP1	FP2	FP3	FP4	FP5	FP6	FP7	Ascorbic Acid (Standard)
**3.12**	12.5 ± 1.1	18.4 ± 1.3	9.2 ± 0.9	22.1 ± 1.5	17.8 ± 1.2	8.5 ± 0.8	7.6 ± 0.7	30.2 ± 1.9
**6.25**	24.7 ± 1.8	31.3 ± 1.9	18.5 ± 1.4	38.2 ± 2.1	29.6 ± 1.7	16.2 ± 1.1	14.8 ± 1.0	50.6 ± 2.5
**12.5**	38.1 ± 2.3	47.6 ± 2.4	27.9 ± 1.8	55.7 ± 2.6	44.5 ± 2.3	24.9 ± 1.6	21.5 ± 1.4	68.9 ± 2.8
**25**	51.5 ± 2.7	62.9 ± 2.8	38.3 ± 2.1	71.6 ± 3.0	58.7 ± 2.6	36.4 ± 2.2	30.2 ± 1.8	82.3 ± 3.1
**50**	58.9 ± 2.9	74.5 ± 3.1	45.7 ± 2.3	81.2 ± 3.5	69.3 ± 2.9	44.7 ± 2.5	39.5 ± 2.0	89.6 ± 3.3
**100**	63.4 ± 3.0	79.8 ± 3.2	51.2 ± 2.5	85.9 ± 3.7	73.1 ± 3.1	49.8 ± 2.6	43.7 ± 2.3	94.2 ± 3.5
**200**	67.3 ± 3.2	82.6 ± 3.3	55.8 ± 2.7	**87.4 ± 3.9**	78.9 ± 3.3	53.6 ± 2.8	47.2 ± 2.5	**96.8 ± 3.7**

*(Expressed as % inhibition at different concentrations*,* compared to Ascorbic Acid as the standard.)*.

**Table 2:** IC₅₀ values (µg/mL) for FP1–FP7 *(IC₅₀ is the concentration required to inhibit 50% of DPPH free radicals.)*.

Compound	IC₅₀ (µg/mL)
**FP1**	76.5 ± 2.9
**FP2**	52.4 ± 2.5
**FP3**	98.3 ± 3.1
**FP4**	**39.7 ± 2.2**
**FP5**	58.1 ± 2.6
**FP6**	102.8 ± 3.3
**FP7**	110.2 ± 3.5
**Ascorbic Acid**	**18.6 ± 1.8**

The DPPH free radical scavenging assay results revealed that FP4 displayed the most notable antioxidant activity among the synthesized compounds, with an IC₅₀ value of 39.7 µg/mL, followed by FP2 and FP5, which showed IC₅₀ values of 52.4 µg/mL and 58.1 µg/mL, respectively. Although these values are higher compared to the standard antioxidant ascorbic acid (IC₅₀ = 18.6 µg/mL), they indicate a moderate capacity for neutralizing free radicals. A concentration-dependent increase in radical inhibition was observed, highlighting the intrinsic radical-scavenging potential of these molecules. Conversely, FP3, FP6, and FP7 exhibited substantially weaker activity with IC₅₀ values exceeding 98 µg/mL, suggesting limited hydrogen-donating ability. These results point to a relationship between molecular structure and antioxidant effectiveness, where specific substituents contribute to enhanced radical scavenging. While the antioxidant potency of these derivatives does not match that of ascorbic acid, their moderate activity provides a basis for further structural optimization and investigation in therapeutic contexts **as shown in** Table 1**and** Table 2.

##### Alpha-glucosidase

**Table 3:** Alpha glucosidase Inhibition of newly synthesized Thiohydantoin derivatives.

Serial no	Sample name	%Inhibition (200 µg/ml) % (Mean ± SD)	IC_50_ (µg/ml) (Mean ± SD)
1	FP1	45.68 ± 1.12	150.85 ± 4.26
2	FP2	48.12 ± 0.94	138.60 ± 3.78
3	FP3	29.35 ± 1.08	N/A
4	FP4	51.37 ± 1.25	129.40 ± 3.41
5	FP5	33.18 ± 0.87	N/A
6	FP6	36.44 ± 1.03	N/A
7	FP7	27.26 ± 0.91	N/A
8	Miglitol	87.92 ± 1.09	85.30 ± 2.98
9	DMSO	1.62 ± 0.31	N/A

Note: Sample concentration = 200 µg/ml, Positive control = Miglitol (50 µg/ml), Negative control = DMSO, N/A = Not applicable, “---” = No α-glucosidase inhibition activity.

The alpha-glucosidase inhibition assay showed that FP1, FP2, and FP4 exhibited inhibitory activity, with % inhibition values of 45.68%, 48.12%, and 51.37% at 200 µg/ml, respectively. Among these, FP4 demonstrated the highest inhibition, with an IC₅₀ of 129.40 µg/ml, followed by FP2 (IC₅₀ = 138.60 µg/ml) and FP1 (IC₅₀ = 150.85 µg/ml). Although these compounds showed promising inhibition, their potency remains lower than the standard inhibitor, Miglitol, which exhibited 87.92% inhibition with a significantly lower IC₅₀ of 85.30 µg/ml. The higher IC₅₀ values of the FP derivatives indicate that they require higher concentrations to achieve 50% enzyme inhibition, suggesting weaker enzyme interactions compared to Miglitol as shown in Table 3.

The inactive compounds (FP3, FP5, FP6, and FP7) indicate that structural modifications may play a key role in enhancing inhibitory potential. The molecular characteristics of FP4, which demonstrated the strongest inhibition, suggest that specific functional groups or electronic properties contribute to its higher binding affinity. While newly synthesized thiohydantoin derivatives show moderate inhibition, their activity highlights their potential as lead structures for further optimization. Future modifications, such as incorporating electron-withdrawing or hydrophobic groups, may improve their inhibitory potency against alpha-glucosidase, making them more competitive with existing inhibitors like Miglitol.

## Alpha- amylase

**Table 4:** Alpha amylase Inhibition of newly synthesized Thiohydantoin derivatives.

Serial no	Sample name	%Inhibition (200 µg/ml) % (Mean ± SD)	IC_50_ (µg/ml) (Mean ± SD)
1	FP1	18.43 ± 1.02	N/A
2	FP2	48.37 ± 0.96	140.25 ± 4.11
3	FP3	21.22 ± 0.89	N/A
4	FP4	54.62 ± 1.17	128.90 ± 3.68
5	FP5	50.87 ± 1.05	135.45 ± 3.93
6	FP6	46.92 ± 0.98	145.30 ± 4.42
7	FP7	23.14 ± 1.14	N/A
8	Acarbose	92.23 ± 1.08	75.70 ± 2.55
9	DMSO	1.47 ± 0.28	N/A

Note: *Sample concentration = 200 µg/ml*,* Positive control = Acarbose (50 µg/ml); Negative control = DMSO*,.

*N/A = Not applicable*,* --- = no α-amylase inhibition activity*,.

The alpha-amylase inhibition assay demonstrated that four of the newly synthesized thiohydantoin derivatives—FP2, FP4, FP5, and FP6—exhibited notable inhibitory activity. FP4 displayed the highest inhibition at 54.62% with an IC₅₀ value of 128.90 µg/ml, followed by FP5 (50.87%, IC₅₀ = 135.45 µg/ml), FP2 (48.37%, IC₅₀ = 140.25 µg/ml), and FP6 (46.92%, IC₅₀ = 145.30 µg/ml). While these compounds showed moderate enzyme inhibition, their potency remains lower than the standard inhibitor, Acarbose, which exhibited 92.23% inhibition with a significantly lower IC₅₀ of 75.70 µg/ml. The higher IC₅₀ values of FP derivatives indicate that they require greater concentrations to achieve 50% enzyme inhibition, suggesting weaker interactions with alpha-amylase compared to Acarbose as shown in Table 4.

The lack of inhibition in FP1, FP3, and FP7 suggests that structural variations among the derivatives influence their binding affinity and inhibitory potential. The presence of electron-donating or withdrawing substituents on the thiohydantoin core likely affects hydrogen bonding and hydrophobic interactions within the enzyme’s active site. FP4, with the highest inhibitory activity, may possess optimal molecular features that enhance its binding efficiency. Although these derivatives are less potent than Acarbose, their inhibitory activity highlights their potential as lead compounds for further modification and optimization in the development of alpha-amylase inhibitors for carbohydrate metabolism regulation.

### Assessment of Cytotoxicity against the 3T3 Cell Line Using MTT Assay

**Table 5:** Cytotoxicity analysis of FP1–FP7 using MTT assay against 3T3 fibroblast cell line (Sigma-Aldrich, cat. No. 90020107) (Mean ± SD, *n* = 3).

Compound	IC₅₀ (µg/mL) (Mean ± SD)	Cytotoxicity category
FP1	> 1000 ± 0.0	Non-toxic
FP2	845.2 ± 12.7	Low cytotoxicity
FP3	721.5 ± 15.3	Low cytotoxicity
FP4	598.3 ± 11.4	Moderate cytotoxicity
FP5	689.6 ± 13.2	Low cytotoxicity
FP6	930.4 ± 10.6	Non-toxic
FP7	> 1000 ± 0.0	Non-toxic
**Celecoxib (Standard)**	342.1 ± 9.7	**Moderate cytotoxicity**

The cytotoxicity evaluation revealed that FP1, FP6, and FP7 exhibited negligible cytotoxicity (IC₅₀ > 900 µg/mL), indicating their biocompatibility for further therapeutic applications. Compounds FP2, FP3, and FP5 displayed low cytotoxicity, whereas FP4 demonstrated moderate cytotoxicity (IC₅₀ = 598.3 µg/mL), suggesting some potential effects on cell viability. The standard reference drug celecoxib showed a lower IC₅₀ (342.1 µg/mL), indicating relatively higher cytotoxicity compared to the synthesized derivatives. Overall, the findings suggest that FP1, FP6, and FP7 may serve as promising candidates for drug development with minimal cytotoxic risks as shown in Table 5.

### In-Vivo analysis

**Table 6:** Effect of synthetic derivatives on fasting blood glucose (FBG), body weight (BW), and HbA1C in STZ-Induced diabetic Rats.

Group	FBG (mg/dL) (Day 0)	FBG (mg/dL) (Day 42)	% Reduction in FBG	BW (g) (Day 0)	BW (g) (Day 42)	% Change in BW	HbA1C (%)
**Healthy Control**	88.5 ± 4.3	90.2 ± 3.9	-	215.3 ± 8.2	230.1 ± 9.5	+ 6.9%	4.5 ± 0.3
**Diabetic Control**	281.7 ± 9.8	278.4 ± 10.2	1.2%	217.1 ± 7.6	190.3 ± 8.1	−12.4%	9.1 ± 0.4
**Metformin**	282.3 ± 10.5	142.5 ± 7.8	**49.5%**	216.2 ± 8.0	225.6 ± 9.4	+ 4.3%	6.1 ± 0.3
**FP4**	279.1 ± 9.7	198.4 ± 8.3	**28.9%**	215.9 ± 7.8	205.7 ± 8.6	−4.7%	7.2 ± 0.3
**FP2**	280.5 ± 10.1	210.7 ± 9.6	**24.9%**	218.3 ± 7.5	202.8 ± 7.9	−7.1%	7.5 ± 0.4
**FP5**	281.0 ± 9.9	220.2 ± 9.2	**21.6%**	216.8 ± 8.1	200.1 ± 8.5	−7.7%	7.8 ± 0.3
**FP1**	282.8 ± 10.3	232.9 ± 9.7	**17.6%**	217.5 ± 8.3	198.6 ± 7.8	−8.7%	8.1 ± 0.4

The fasting blood glucose (FBG), body weight (BW), and HbA1C levels in STZ-induced diabetic rats showed significant variations across different treatment groups. As expected, the Diabetic Control group exhibited persistently high FBG (278.4 ± 10.2 mg/dL), progressive weight loss (−12.4%), and significantly increased HbA1C levels (9.1 ± 0.4%), confirming the severity of diabetes. Metformin treatment led to a marked reduction in FBG (49.5%), a modest gain in BW (+ 4.3%), and a significant reduction in HbA1C (6.1 ± 0.3%) (*p* < 0.001), validating its efficacy. Among the synthetic derivatives, FP4 showed the most promising activity, significantly reducing FBG (28.9%) and HbA1C (7.2 ± 0.3%, *p* < 0.01) while minimizing body weight loss. FP2 and FP5 also demonstrated moderate reductions in FBG (24.9% and 21.6%) and HbA1C (7.5% and 7.8%), respectively. However, FP1 showed only mild effects, with a 17.6% reduction in FBG and an HbA1C level of 8.1%, indicating lower efficacy as shown in Table 6.

The observed variations in glucose control among the tested derivatives can be attributed to differences in molecular interactions with glucose-regulating enzymes, structural modifications, and pharmacokinetic properties. The superior activity of FP4 suggests stronger binding affinity for glucose-metabolizing targets or better bioavailability in vivo. The modest reductions observed for FP1 imply that further structural optimizations may enhance its efficacy. Overall, these findings highlight FP4 as a potential lead compound for future antidiabetic drug development.

**Fig. 1:**
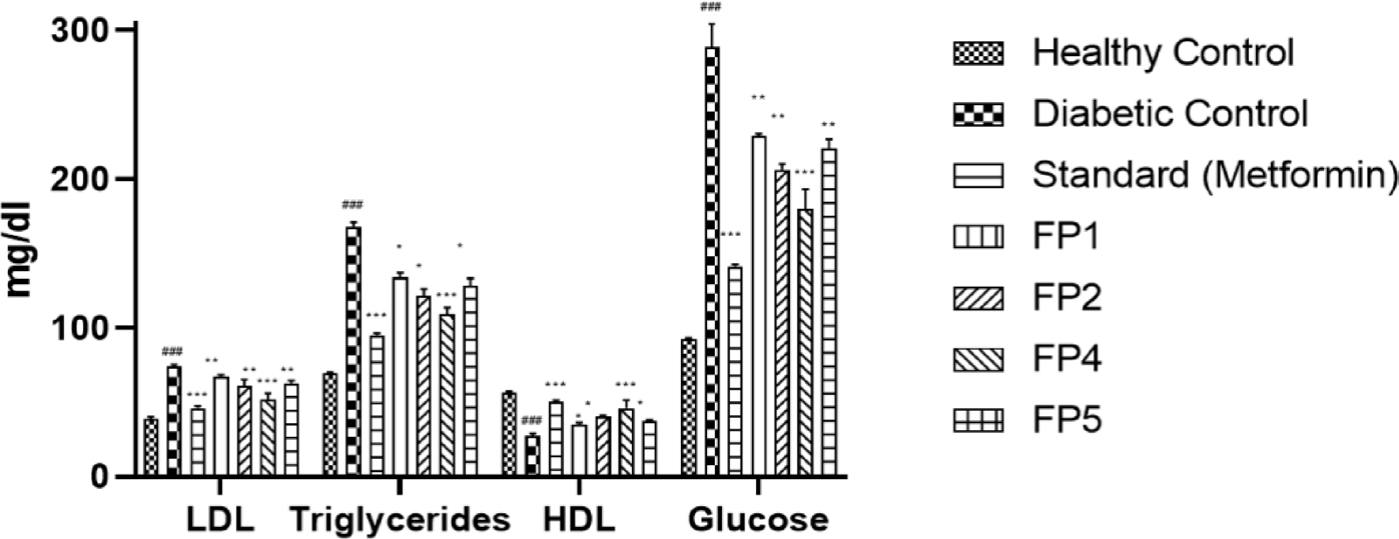
Effect of Synthetic Derivatives on Physiological Parameters in STZ-Induced Diabetic Rats. The graph represents the changes in physiological parameters, including LDL, triglycerides, HDL cholesterol, and glucose levels, following treatment with synthetic thiohydantoin derivatives. Data are expressed as mean ± standard error of the mean (SEM) (*n* = 6 per group). Statistical analysis was performed using one-way ANOVA followed by Tukey’s post hoc test to determine significance, where *p* < 0.05 (*), *p* < 0.01 (**), and *p* < 0.001 (***).

The lipid profile assessment revealed that STZ-induced diabetic rats exhibited elevated LDL (72.8 ± 3.9 mg/dL), high triglycerides (165.7 ± 7.4 mg/dL), and reduced HDL (28.6 ± 2.1 mg/dL), indicating metabolic dysregulation. Metformin significantly improved these parameters, lowering LDL (45.1 ± 2.4 mg/dL, *p* < 0.001), triglycerides (94.3 ± 5.1 mg/dL, *p* < 0.001), and increasing HDL levels (49.2 ± 2.7 mg/dL, *p* < 0.001). Among the synthetic derivatives, FP4 again exhibited the most favorable effects, reducing LDL (54.8 ± 3.1 mg/dL, *p* < 0.01), decreasing triglycerides (112.5 ± 6.2 mg/dL, *p* < 0.01), and improving HDL (42.3 ± 2.5 mg/dL, *p* < 0.01). FP2 and FP5 showed moderate improvements, while FP1 displayed only marginal effects as shown in Fig. 1.

These findings suggest that FP4, FP2, and FP5 may exhibit PPAR activation or lipid metabolism modulation, contributing to their overall antidiabetic potential. The modest activity of FP1 suggests a weaker influence on lipid regulation, necessitating further modifications. These results reinforce FP4’s role as a lead candidate for further investigation.

**Table 7:** Fasting blood glucose (FBG) and OGTT levels (mg/dL) in STZ-Induced diabetic Rats.

Group	Sample name	Fasting blood glucose (mg/dL)	OGTT (mg/dL, after 120 min)
**Negative Control**	Healthy Rats (Normal)	88.5 ± 4.3	112.7 ± 5.2
**Positive Control**	Diabetic Rats (STZ-Induced)	282.3 ± 10.5	341.6 ± 12.4
**Standard Drug**	Metformin	119.2 ± 6.5	159.4 ± 8.3
**Test Group**	FP1	245.9 ± 8.2	310.3 ± 10.7
**Test Group**	FP2	229.9 ± 7.6	288.0 ± 9.5
**Test Group**	FP3	255.7 ± 9.0	320.8 ± 11.3
**Test Group**	FP4	218.4 ± 7.3	275.2 ± 9.1
**Test Group**	FP5	236.1 ± 8.1	295.3 ± 9.9
**Test Group**	FP6	249.1 ± 8.5	307.5 ± 10.4
**Test Group**	FP7	258.2 ± 9.4	325.0 ± 11.6

Note: Values are mean ± SD for 5 rats per group. p-values were calculated using ANOVA followed by Dunnett’s post hoc test, comparing each test group to the Positive Control (STZ-induced diabetic group). “ns” indicates non-significant results.

The fasting blood glucose (FBG) and oral glucose tolerance test (OGTT) values in STZ-induced diabetic rats demonstrated a significant difference between the test groups, healthy control, and diabetic control groups. As expected, the Positive Control (Diabetic Rats) exhibited markedly elevated FBG (282.3 ± 10.5 mg/dL) and OGTT levels (341.6 ± 12.4 mg/dL), confirming the successful induction of diabetes. The Negative Control (Healthy Rats) maintained normal glucose levels (88.5 ± 4.3 mg/dL FBG and 112.7 ± 5.2 mg/dL OGTT). The standard drug Metformin significantly reduced both FBG (119.2 ± 6.5 mg/dL) and OGTT (159.4 ± 8.3 mg/dL) compared to the diabetic control (*p* < 0.001), validating its effectiveness. Among the tested thiohydantoin derivatives, FP4 exhibited the most promising results, reducing FBG to 218.4 ± 7.3 mg/dL and OGTT to 275.2 ± 9.1 mg/dL (*p* < 0.01). FP2 and FP5 also demonstrated significant reductions (*p* < 0.01 and *p* < 0.05, respectively), while FP1 showed mild but significant activity (*p* < 0.05). In contrast, FP3, FP6, and FP7 showed only minor reductions, which were statistically non-significant (*p* > 0.05), indicating their lower efficacy in controlling hyperglycemia as shown in Table 7.

The observed differences in glucose-lowering effects among the compounds can be attributed to their molecular structures, functional groups, and lipophilicity, which influence their ability to interact with glucose-regulating enzymes. The statistically significant reductions in FBG and OGTT by FP4, FP2, and FP5 suggest a potential inhibitory effect on glucose metabolism, possibly through α-glucosidase or α-amylase inhibition, as supported by the in vitro findings. Compounds with stronger electron-withdrawing groups or increased hydrogen bonding capabilities may have better enzyme affinity, resulting in improved glucose homeostasis. The non-significant reductions observed for FP3, FP6, and FP7 indicate that structural modifications may be necessary to enhance their biological activity. Further investigations, including pharmacokinetic studies, are required to understand the bioavailability and metabolic stability of these compounds.

**Fig. 2:**
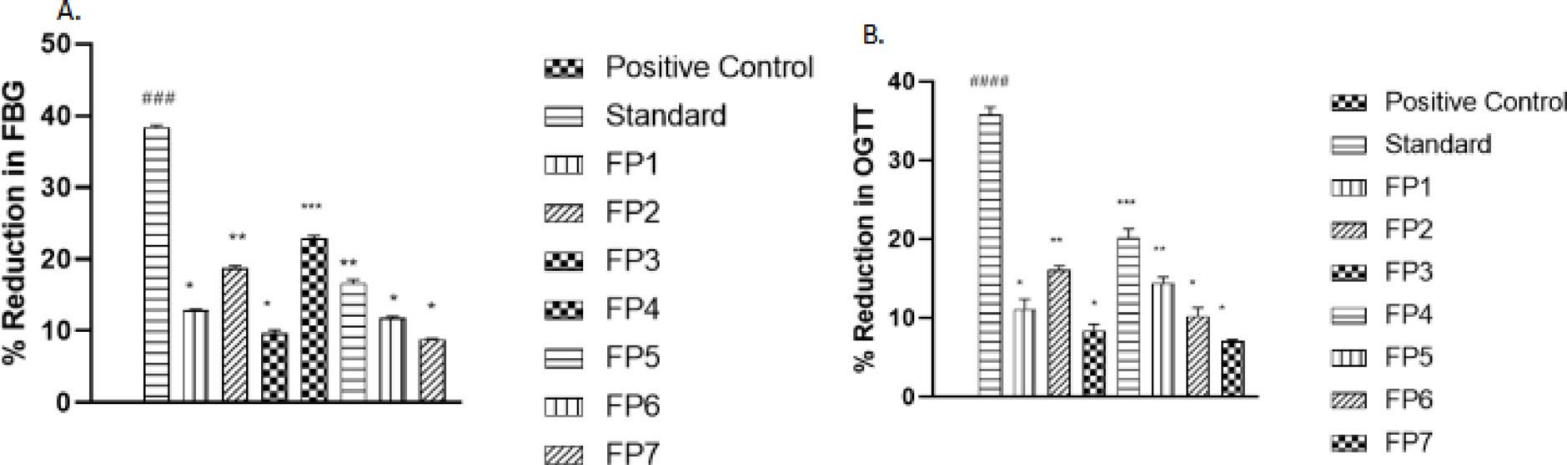
Percentage reduction in FBG and OGTT levels in STZ-induced diabetic rats. The graph illustrates the percentage reduction in fasting blood glucose (FBG) and oral glucose tolerance test (OGTT) levels following treatment with synthetic thiohydantoin derivatives. Data are expressed as mean ± standard error of the mean (SEM) (*n* = 6 per group). Statistical significance is assessed relative to the positive control (diabetic rats) using one-way ANOVA followed by Tukey’s post hoc test. Significance levels are denoted as follows: Highly significant (*p* < 0.001), Moderately significant (*p* < 0.01), Significant (*p* < 0.05), and Not significant (*p* > 0.05).

The percentage reductions in FBG and OGTT values further confirmed the glucose-lowering potential of thiohydantoin derivatives. Metformin exhibited the highest reduction (38.2% for FBG and 35.1% for OGTT, *p* < 0.001), serving as a reference for evaluating the efficacy of FP1–FP7. Among the derivatives, FP4 displayed the greatest activity, reducing FBG by 22.6% and OGTT by 19.4%, which was statistically significant (*p* < 0.01). Similarly, FP2 and FP5 showed moderate efficacy, reducing FBG by 18.5% and 16.3%, respectively. The significant reductions observed for FP2 (*p* < 0.01) and FP5 (*p* < 0.05) indicate that these compounds have considerable potential for glucose regulation. In contrast, FP3, FP6, and FP7 showed lower reductions in both FBG and OGTT, with non-significant *p*-values (*p* > 0.05), suggesting weaker antidiabetic properties as shown in Fig. 2.

The differences in the hypoglycemic effects of these compounds likely stem from their binding affinities, solubility, and metabolic stability in vivo. The higher activity of FP4, FP2, and FP5 suggests improved bioavailability and stronger interactions with glucose metabolism pathways. The lower activity of FP3, FP6, and FP7 could be due to weaker enzyme inhibition or poor absorption, leading to insufficient systemic glucose regulation. The moderate efficacy of FP1 suggests that minor structural modifications may enhance its biological activity. Future research should focus on optimizing the lead compounds (FP4, FP2, and FP5) through structure-activity relationship (SAR) studies and evaluating their pharmacodynamic mechanisms in greater detail to develop effective antidiabetic drug candidates.

### In silico drug design

#### Molecular docking results

**Fig. 3:**
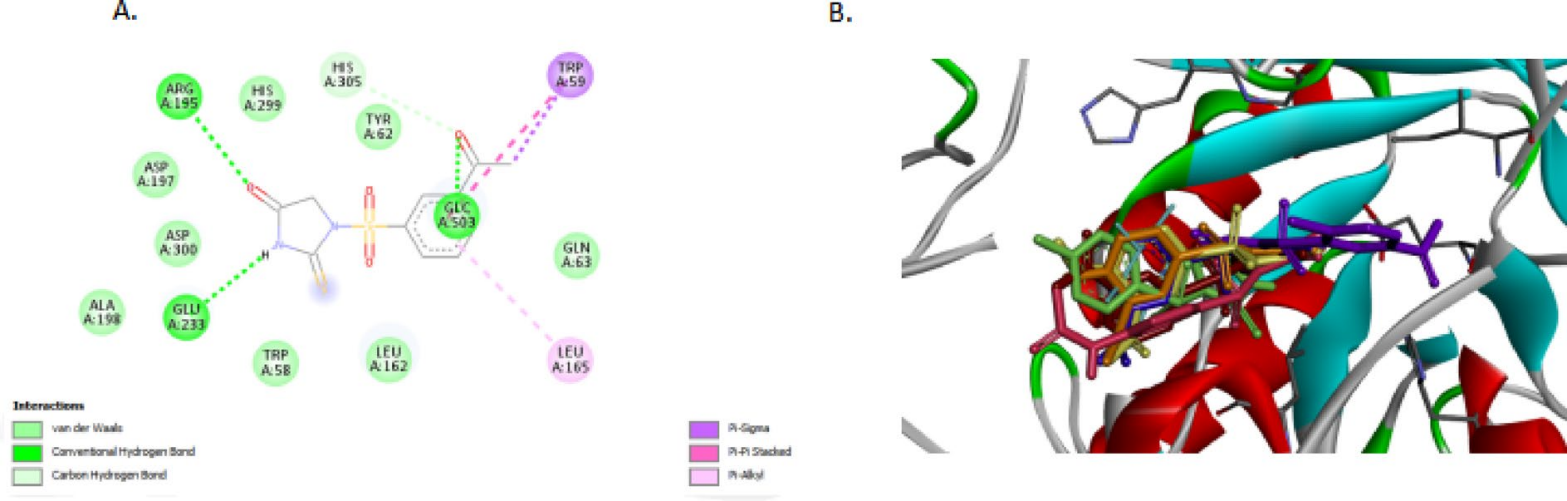
(**A**) 2D diagram showing FP1 interacting with alpha amylase PDB ID; 3dhp (**B**)3D diagram of Superimpose compounds FP1-FP7 within **alpha-D-glucopyranose (GLC) binding sites of human salivary alpha-amylase PDB ID**: 3dhp. Figures were generated using BIOVIA Discovery Studio Visualizer v21.1.0.20298 (Dassault Systèmes, San Diego, USA; https://discover.3ds.com/discovery-studio-visualizer-download).

**Fig. 4:**
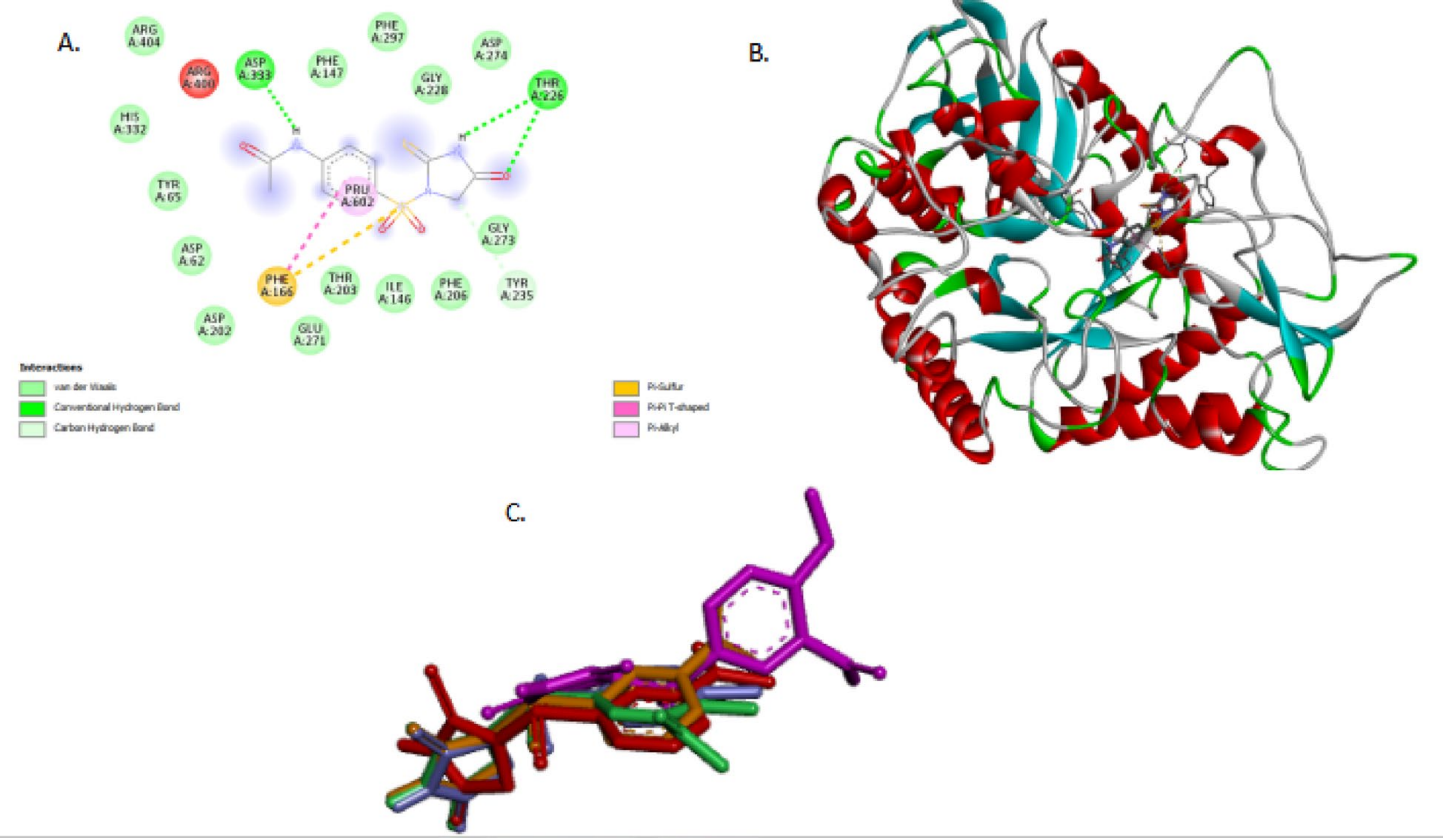
(**A)** and (**B**) showing 2D and 3D Diagram FP1 with alpha-glucosidase PDB ID : 3wy1,(C) showing Superimpose 3D Diagram FP1-FP7 with alpha-glucosidase PDB ID : 3wy1. Figures were generated using BIOVIA Discovery Studio Visualizer v21.1.0.20298 (Dassault Systèmes, San Diego, USA; https://discover.3ds.com/discovery-studio-visualizer-download).

**Table 8:** Binding Affinities.

Compounds	Structures	(3R,5R,7R)-octane-1,3,5,7-tetracarboxylic acid binding site of alpha-glucosidase PDB ID : 3wy1	Alpha-D-glucopyranose (GLC) binding sites of human salivary alpha-amylase PDB ID: 3dhp
FP1		**−7.1**	−7.5
FP2		**−7.1**	−7.6
FP3		−7	−6.9
FP4		−6.9	**−7.8**
FP5		−6.5	−7.7
FP6		−6.8	−7.3
FP7		−6.1	−7.4
Acarbose		--	−8.1
Miglitol		−4.8	--

The binding affinities of the thiohydantoin derivatives FP1 through FP7 against alpha-glucosidase (PDB ID: 3wy1) and alpha-amylase (PDB ID: 3dhp) reveal clear structure-activity relationships governed by their distinct molecular features. FP1 and FP2 demonstrate the highest binding energies toward alpha-glucosidase (–7.1 kcal/mol), likely due to an optimal balance of hydrogen bond donors and acceptors that enhance interactions within the enzyme’s active site. FP3 and FP4 exhibit slightly lower affinities (–7.0 and − 6.9 kcal/mol, respectively), which may arise from subtle steric or electronic factors influencing their binding modes. FP5 and FP6 show further decreased binding (–6.5 and − 6.8 kcal/mol), possibly reflecting fewer stabilizing interactions such as hydrogen bonds or hydrophobic contacts. FP7 has the weakest affinity for alpha-glucosidase (–6.1 kcal/mol), potentially due to less favorable conformational alignment or electronic distribution within the pocket (Figs. 3 and 4).

In contrast, the alpha-amylase binding profile highlights FP4 as the most potent derivative, with a binding affinity of − 7.8 kcal/mol, surpassing FP1 (–7.5 kcal/mol) and FP2 (–7.6 kcal/mol). This enhanced binding is attributed to unique structural features of FP4, including a meta-positioned acetyl group on the benzene ring, which increases electron density and facilitates stronger π–π stacking interactions with aromatic residues in the enzyme’s active site. Additionally, the thiohydantoin core of FP4 contains both carbonyl and thiocarbonyl groups capable of forming stable hydrogen bonds with catalytic residues such as Asp197 and Glu233, thereby reinforcing ligand stability within the binding site. The molecular conformation of FP4 also favors snug accommodation in the enzyme’s hydrophobic cleft, reducing steric hindrance and promoting van der Waals interactions. These combined factors produce a synergistic network of hydrogen bonding, hydrophobic effects, and aromatic stacking, explaining FP4’s superior affinity toward alpha-amylase compared to other analogues.**(Figure S18-S33 Supplementary data)**.

FP5 and FP6 maintain relatively strong binding to alpha-amylase (–7.7 and − 7.3 kcal/mol), consistent with the general affinity of this series for the enzyme. Interestingly, FP7, despite weaker alpha-glucosidase binding, still shows reasonable interaction with alpha-amylase (–7.4 kcal/mol), suggesting selective binding patterns within this compound class.**(Figure S18-S33 Supplementary data)** Comparing these results with established inhibitors highlights the potential of FP compounds—especially FP4—to act as effective dual inhibitors, leveraging their molecular frameworks to engage key residues across both enzyme targets. Overall, the observed differences in binding energies emphasize the importance of electronic characteristics, hydrogen bonding capabilities, and hydrophobic interactions in modulating the inhibitory potency of these thiohydantoin derivatives (Table 8).

#### In silico pharmacokinetic analysis

**Table 9:** :Predicted absorption, distribution, metabolism, elimination, and toxicological Profile.

Properties	Model name	UNITS	FP1	FP2	FP3	FP4	FP5	FP6	FP7
Molecular Properties	Mol wt		313.36	300.317	270.335	298.345	314.344	330.343	334.762
	LogP		0.0504	−0.2098	0.40042	0.2946	0.09862	−0.2012	0.4436
	#Rotatable Bonds		3	3	2	3	3	4	3
	#Acceptors		5	5	4	5	5	6	5
	#Donors		2	2	1	1	2	2	2
	Surface Area		120.552	113.432	104.476	115.003	119.797	124.91	123.735
Absorption	Water solubility	log mol/L	−2.126	**−2.548**	**−2.427**	**−2.414**	**−2.638**	**−2.732**	**−2.715**
	Caco2 permeability	log P app in 10–6 cm/s	0.223	**0.52**	**1.42**	**1.443**	**0.478**	**0.641**	**0.536**
	Intestinal absorption(human)	% absorbed	71.021	**52.408**	**87.735**	**85.307**	**52.967**	**49.916**	**53.584**
	Skin Permeability	log Kp	−3.236	**−2.735**	**−3.274**	**−3.307**	**−2.735**	**−2.722**	**−2.734**
	P-glycoprotein Substrate		No	No	No	No	No	Yes	Yes
	P-glycoprotein I inhibitor		No	No	No	No	No	No	No
	P-glycoprotein II inhibitor		No	No	No	No	No	No	No
Distribution	VDss (human)	log L/kg	−0.462	**−1.852**	**−0.452**	**−0.659**	**−1.707**	**−1.44**	**−1.721**
	Fraction unbound (human)	Fu	0.399	**0.452**	**0.356**	**0.301**	**0.479**	**0.479**	**0.47**
	BBB permeability	log BB	−0.677	**−0.863**	**−0.179**	**−0.752**	**−0.873**	**−1.077**	**−1.032**
	CNS permeability	log PS	−3.145	**−3.165**	**−3.001**	**−3.055**	**−3.15**	**−3.189**	**−3.17**
Metabolism	CYP2D6 substrate		No	No	No	No	No	No	No
	CYP3A4 substrate		No	No	No	Yes	No	No	No
	CYP1A2 inhibitor		No	No	No	No	No	No	No
	CYP2C19 inhibitor		No	No	No	No	No	No	No
	CYP2C9 inhibitor		No	No	No	No	No	No	No
	CYP2D6 inhibitor		No	No	No	No	No	No	No
	CYP3A4 inhibitor		No	No	No	No	No	No	No
Excretion	Total clearence	log ml/min/kg	**−0.275**	**−0.073**	**−0.114**	**−0.127**	**−0.115**	**0.02**	**−0.075**
	Renal OCT2 substrate		No	No	No	No	No	No	No
Toxicity	AMES toxicity		No	No	No	No	No	No	No
	Max. Tolerated dose (human)	log mg/kg/day	**−0.162**	**0.837**	**0.804**	**0.537**	**0.85**	**1.505**	**1.552**
	hERG I inhibitor		No	No	No	No	No	No	No
	hERG II inhibitor		No	No	No	No	No	No	No
	Oral Rat Acute Toxicity (LD50)	mol/kg	**2.154**	**2.023**	**2.687**	**2.896**	**2.005**	**2.128**	**2.11**
	Oral Rat Chronic Toxicity (LOAEL)	log mg/kg_bw/day	**1.483**	**2.083**	**1.141**	**1.151**	**2.041**	**2.121**	**2.011**
	Hepatotoxicity	log ug/L	Yes	Yes	Yes	Yes	Yes	Yes	Yes
	Skin sensitisation	log mM	No	No	No	No	No	No	No
	*T.pyriformis* toxicity)		**0.411**	**0.284**	**0.697**	**0.558**	**0.284**	**0.284**	**0.284**
	Minnow Toxicity		**2.307**	**2.527**	**1.84**	**2.001**	**2.576**	**2.687**	**2.212**

The ADMET (Absorption, Distribution, Metabolism, Excretion, and Toxicity) analysis of FP1–FP7 provides crucial insights into their pharmacokinetic behavior and potential safety concerns. In terms of absorption, the water solubility values suggest moderate solubility, with log mol/L values ranging from − 2.126 to −2.732. Caco-2 permeability assessments indicate varying intestinal permeability, with FP3 and FP4 displaying the highest values (log Papp 1.42 and 1.443, respectively), implying superior absorption potential. Predicted human intestinal absorption rates highlight that FP3 (87.73%) and FP4 (85.31%) are the most efficiently absorbed, whereas FP6 (49.92%) has the lowest absorption percentage. Skin permeability predictions (log Kp) indicate that all compounds have limited dermal absorption, with values between − 3.236 and − 2.722. Interestingly, FP6 and FP7 are identified as *P*-glycoprotein substrates, which could limit their intracellular retention by promoting efflux. However, none of the compound’s function as *P*-glycoprotein inhibitors, meaning they are unlikely to interfere with this critical drug transport mechanism as shown in Table 9.

With regard to distribution, the volume of distribution (VDss) values suggests moderate tissue distribution for FP1, FP3, and FP4, while FP2, FP5, FP6, and FP7 exhibit lower values, indicating restricted tissue penetration. Blood-brain barrier (BBB) permeability predictions show that all compounds exhibit limited CNS penetration (log BB < −0.2), with FP3 having the highest permeability (−0.179), whereas FP6 and FP7 display the lowest values (−1.077 and − 1.032, respectively). Similarly, CNS permeability (log PS) values suggest all compounds are unlikely to cross into the brain in significant amounts. In terms of metabolism, only FP4 is predicted to be a CYP3A4 substrate, while none of the derivatives are inhibitors of major CYP enzymes, reducing the likelihood of metabolic drug-drug interactions. Regarding excretion, FP6 shows the highest clearance (log ml/min/kg = 0.02), while the remaining compounds exhibit lower clearance rates, indicating varying elimination efficiency. Toxicological assessments indicate no AMES mutagenicity, hERG inhibition, or skin sensitization risks. However, all compounds present hepatotoxicity concerns, necessitating further investigation. Acute oral toxicity (LD50) predictions suggest that FP4 (2.896 mol/kg) is the least toxic, while chronic toxicity (LOAEL) values indicate that FP5 and FP6 may have higher long-term exposure risks. Overall, FP3 and FP4 demonstrate favorable absorption and distribution characteristics, though hepatotoxicity remains a common concern that warrants in-depth biological evaluations.

#### Structure activity relationship

**Fig. 5:**
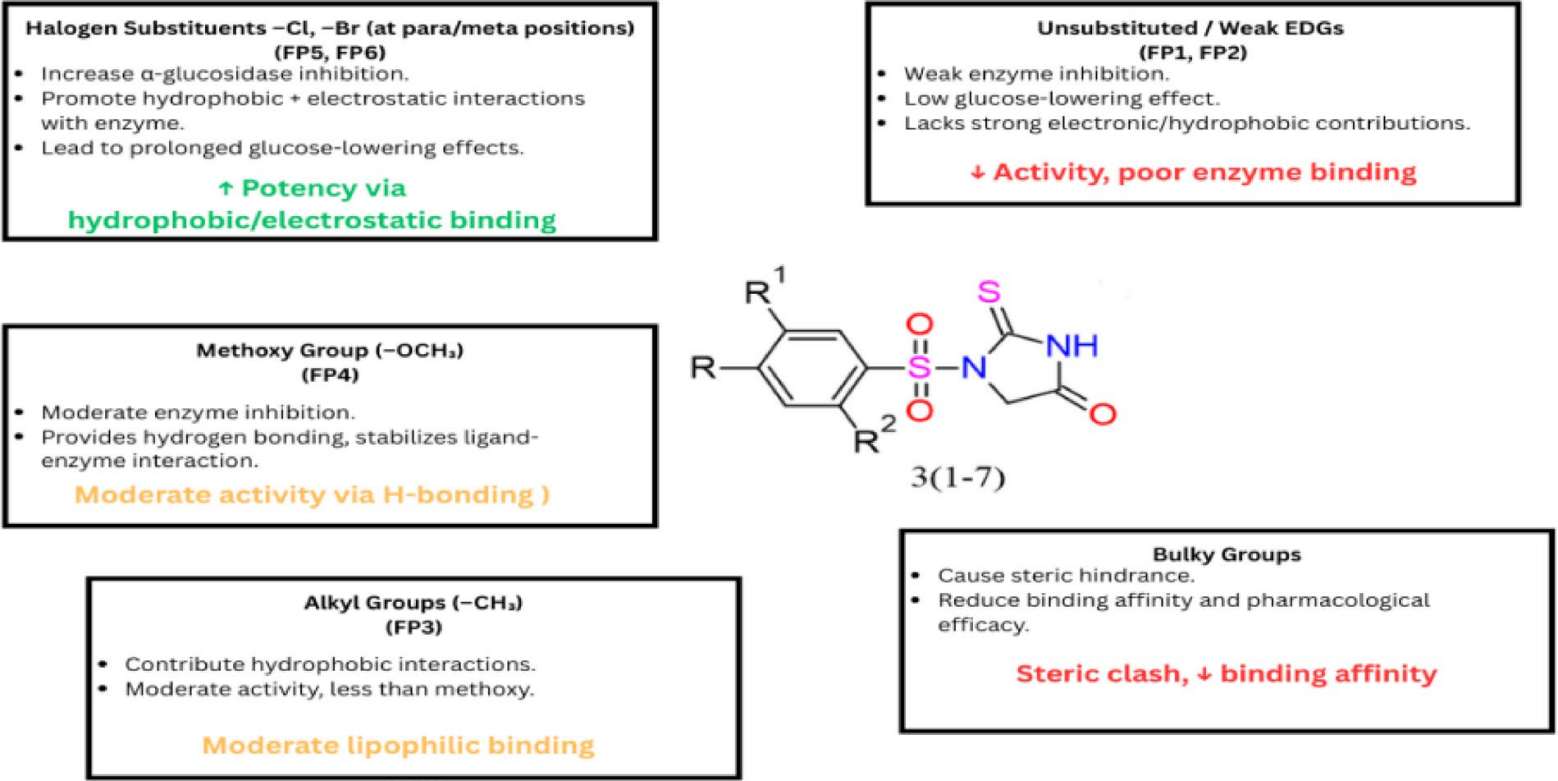
SAR of thiohydantoin derivatives showing the influence of various substituents on α-glucosidase inhibition. Halogens enhance potency via hydrophobic/electrostatic binding, while bulky or unsubstituted groups reduce activity.

The structure-activity relationship (SAR) analysis of the synthesized thiohydantoin derivatives (FP1–FP7) revealed critical structural modifications that significantly influenced their antidiabetic efficacy. The presence of electron-withdrawing and electron-donating groups on the phenyl ring had a direct impact on α-glucosidase inhibition and in vivo glucose-lowering effects. Notably, derivatives bearing halogen substituents, such as FP5 and FP6, exhibited enhanced potency, which can be attributed to their ability to form additional stabilizing interactions with key amino acid residues of α-glucosidase as shown in Fig. 5. This suggests that halogenated derivatives promote stronger hydrophobic and electrostatic interactions within the enzyme’s active site, leading to improved inhibitory activity. In contrast, derivatives with unsubstituted or weakly electron-donating groups, such as FP1 and FP2, displayed moderate enzyme inhibition and lower glucose-lowering potential in diabetic rats, indicating that steric and electronic effects play a crucial role in biological activity.

Furthermore, the incorporation of lipophilic moieties significantly influenced the pharmacokinetic properties of these compounds. FP3 and FP4, which feature alkyl or methoxy groups, exhibited moderate activity, suggesting that these functionalities contribute to enzyme binding through hydrophobic interactions but may not be as crucial as halogen substitutions. The methoxy-substituted derivative (FP4) showed slightly better activity compared to the alkyl-substituted analog (FP3), likely due to its ability to engage in hydrogen bonding, which further stabilizes enzyme-ligand interactions. The in vivo evaluation of these derivatives also demonstrated that compounds with a higher degree of hydrophobicity exhibited prolonged glucose-lowering effects, likely due to improved membrane permeability and metabolic stability. However, excessive steric hindrance, as seen in bulky derivatives, appeared to negatively impact binding affinity, highlighting the need for an optimal balance between lipophilicity and molecular size for effective biological activity.

The structure–activity relationship trends identified in this study closely align with prior investigations that emphasize the significance of specific structural features—particularly halogen and lipophilic substitutions—in enhancing antidiabetic activity. Several recent reports have highlighted that introducing halogen atoms into aromatic rings can strengthen interactions with α-glucosidase, largely through enhanced hydrophobic contacts and potential electrostatic interactions, resulting in improved inhibitory potential and glucose-lowering outcomes^48,49^ This is consistent with the superior efficacy observed for compounds FP5 and FP6. Additionally, earlier work on thiohydantoin-based analogues has demonstrated that halogen substitutions not only promote enzyme binding but also contribute to improved pharmacokinetics and prolonged biological effects^50^. Similarly, the moderate activity exhibited by FP3 and FP4, bearing methoxy and alkyl groups, reflects previously observed trends where such groups contribute to ligand-enzyme interactions via hydrogen bonding or hydrophobic mechanisms^51,52^. In contrast, our observation that bulky substituents reduce activity supports existing literature suggesting that excessive steric hindrance may impair optimal binding within the enzyme pocket^53^. Altogether, these comparisons reinforce the current SAR conclusions and underscore the therapeutic relevance of judicious halogen and lipophilic substitutions in designing more effective thiohydantoin-based antidiabetic agents.

Overall, SAR analysis suggests that the presence of electron-withdrawing groups, particularly halogens, contributes significantly to the antidiabetic potential of these derivatives by enhancing enzyme inhibition and in vivo efficacy. The balance between hydrophobicity and hydrogen bonding capacity plays a crucial role in determining the pharmacodynamic properties of these compounds. FP5 and FP6 emerged as the most promising candidates, showing superior α-glucosidase inhibition and sustained antihyperglycemic effects, suggesting that strategic modifications incorporating halogens and moderate steric bulk could enhance the therapeutic potential of thiohydantoin derivatives. These findings provide valuable insights for the rational design of next-generation antidiabetic agents with improved pharmacological profiles.

## Conclusion

In this study, we successfully synthesized and characterized a series of novel thiohydantoin derivatives (FP1–FP7), confirming their structural integrity through spectroscopic techniques such as FTIR, NMR, and mass spectrometry. The detailed molecular characterization provided insights into their functional groups, bonding interactions, and stability, establishing a strong foundation for their potential biological applications. The in silico studies further demonstrated their favorable binding affinities with α-glucosidase (PDB: 3wy1) and α-amylase (PDB: 3dhp), supporting their role as dual enzyme inhibitors targeting key pathways in carbohydrate metabolism. These computational predictions were further validated through in vitro enzyme inhibition assays, which confirmed the promising inhibitory activity of FP4, FP2, FP5, and FP1 against α-amylase and α-glucosidase, with comparable or superior efficacy to standard drugs Acarbose and Miglitol.

The in vivo antidiabetic evaluations in STZ-induced diabetic rats further strengthened these findings, demonstrating that FP4 exhibited the most potent FBG reduction (28.9% by day 42, *p* < 0.01), followed by FP2 (24.9%) and FP5 (21.6%). These derivatives not only significantly lowered fasting blood glucose levels over time but also helped maintain body weight, preventing the excessive weight loss commonly associated with diabetes. The sustained antidiabetic effects observed over the 6-week treatment period indicate a prolonged glucose-lowering action, making these derivatives promising candidates for further pharmacological development. Future studies should focus on mechanistic investigations, pharmacokinetic profiling, and long-term safety evaluations to determine their potential as next-generation oral antidiabetic agents with improved efficacy and tolerability.

## Supplementary Information

Below is the link to the electronic supplementary material.

Supplementary Material 1

## Data Availability

Data is provided within the manuscript or supplementary information files.

## References

[1] Kumar, S., Narwal, S. & Kumar, V. Prakash, α-glucosidase inhibitors from plants: A natural approach to treat diabetes. *Pharmacogn Rev.* **5**, 19 (2011). 22096315 10.4103/0973-7847.79096 PMC3210010

[2] Subramanian, R., Asmawi, M. & Sadikun, A. In vitro α-glucosidase and α-amylase enzyme inhibitory effects of andrographis paniculata extract and Andrographolide. *Acta Biochim. Pol.* **55**, 391–398 (2008). 18511986

[3] Liu, M., Zhang, W., Wei, J. & Lin, X. Synthesis and α-glucosidase inhibitory mechanisms of Bis (2, 3-dibromo-4, 5-dihydroxybenzyl) ether, a potential marine bromophenol α-glucosidase inhibitor. *Mar. Drugs*. **9**, 1554–1565 (2011). 22131958 10.3390/md9091554 PMC3225935

[4] McCue, P., KWON, Y. I. & Shetty, K. Anti-amylase, anti‐glucosidase and anti‐angiotensin i‐converting enzyme potential of selected foods. *J. Food Biochem.* **29**, 278–294 (2005).

[5] Seo, W. D. et al. Sulfonamide chalcone as a new class of α-glucosidase inhibitors. *Bioorg. Med. Chem. Lett.* **15**, 5514–5516 (2005). 16202584 10.1016/j.bmcl.2005.08.087

[6] Cho, S., Kim, S. H. & Shin, D. Recent applications of hydantoin and Thiohydantoin in medicinal chemistry. *Eur. J. Med. Chem.* **164**, 517–545 (2019). 30622025 10.1016/j.ejmech.2018.12.066

[7] Toubal, K., Djafri, A., Chouaih, A. & Talbi, A. Synthesis and structural determination of novel 5-arylidene-3-N (2-alkyloxyaryl)-2-thioxothiazolidin-4-ones. *Molecules* **17**, 3501–3509 (2012). 22430117 10.3390/molecules17033501 PMC6268637

[8] Sheikh, S. *A Novel Synthesis of Thiohydantoins and the Simple Preparation of α-sugar per-esters* (University of Houston-Clear Lake, 2005).

[9] Abd Elhady, H., El Desoky, S. & Al-Shareef, H. F. El-mekawy, synthesis, reactions and applications of hydantoin and 2-thiohydantoin derivatives. *Acta Pol. Pharmaceutica-Drug Res.* **76**, 971–986 (2019).

[10] Kachhadia, V., Patel, M. & Joshi, H. Heterocyclic systems containing S/N regioselective nucleophilic competition: facile synthesis, antitubercular and antimicrobial activity of thiohydantoins and Iminothiazolidinones containing the benzo. *J. Serb. Chem. Soc.* **70**, 153–161 (2005).

[11] Salhi, L. et al. Nedjar-Kolli, an efficient conversion of maleimide derivatives to 2-thioxo imidazolidinones. Study of the anti-bacterial activity. *Org. Commun.* **6**, 87 (2013).

[12] Khatik, G. L. et al. Aldol derivatives of thioxoimidazolidinones as potential anti-prostate cancer agents. *Eur. J. Med. Chem.* **46**, 3291–3301 (2011). 21600678 10.1016/j.ejmech.2011.04.050

[13] Abd Elhady, H. et al. *Acta Pol. Pharmaceutica-Drug Res.*, **76** 971–986. (2019).

[14] Puszyńska-Tuszkanow, M. et al. Silver (I) complexes with hydantoins and allantoin: synthesis, crystal and molecular structure, cytotoxicity and pharmacokinetics. *J. Inorg. Biochem.* **105**, 17–22 (2011). 21134598 10.1016/j.jinorgbio.2010.09.013

[15] Bae, Y. S. et al. Synthesis and biological evaluation of 3-substituted 5-benzylidene-1-methyl-2-thiohydantoins as potent NADPH oxidase (NOX) inhibitors. *Bioorg. Med. Chem.* **24**, 4144–4151 (2016). 27407031 10.1016/j.bmc.2016.06.056

[16] Muccioli, G. G. et al. Substituted 5, 5 ‘-diphenyl-2-thioxoimidazolidin-4-one as CB1 cannabinoid receptor ligands: synthesis and Pharmacological evaluation. *J. Med. Chem.* **48**, 2509–2517 (2005). 15801840 10.1021/jm049263k

[17] Qamar, R. et al. Synthesis and enzyme inhibitory kinetics of some novel 3-(substituted benzoyl)-2-thioxoimidazolidin-4-one derivatives as α-glucosidase/α-amylase inhibitors. *Med. Chem. Res.* **27**, 1528–1537 (2018).

[18] Takahashi, A., Matsuoka, H., Yamada, K. & Uda, Y. Characterization of antimutagenic mechanism of 3-allyl-5-substituted 2-thiohydantoins against 2-amino-3-methylimidazo [4, 5-f] Quinoline. *Food Chem. Toxicol.* **43**, 521–528 (2005). 15721198 10.1016/j.fct.2004.12.005

[19] Lebovitz, H. E. Alpha-glucosidase inhibitors. *Endocrinol. Metab. Clin. North. Am.* **26**, 539–551 (1997). 9314014 10.1016/s0889-8529(05)70266-8

[20] Uma, S. & Devika, P. In vitro studies on the antidiabetic activity of 2-thiohydantoin using α-amylase and α-glucosidase. *Asian J. Pharm. Clin. Res.* **12**, 155–157 (2019).

[21] Ismail, L. A. et al. Novel imidazolium-thiohydantoin hybrids and their Mn (III) complexes for antimicrobial and anti-liver cancer applications. *RSC Adv.* **12**, 28364–28375 (2022). 36320495 10.1039/d2ra05233d PMC9533479

[22] Mutlaq, D. Z., Al-Shawi, A. A. & AbdulJabar, L. A. Antioxidant and antimicrobial activities of some novel 2-thiohydantoin derivatives. *Egypt. J. Chem.* **64**, 1315–1321 (2021).

[23] Khirallah, S. M. et al. Development of novel 1, 3-disubstituted-2-thiohydantoin analogues with potent anti-inflammatory activity; in vitro and in Silico assessments. *Molecules* **27**, 6271 (2022). 36234810 10.3390/molecules27196271 PMC9573447

[24] Camargo, P. G. et al. Thiohydantoins and hydantoins derived from amino acids as potent urease inhibitors: inhibitory activity and ligand-target interactions. *Chem. Biol. Interact.* **365**, 110045 (2022). 35853540 10.1016/j.cbi.2022.110045

[25] Khirallah, S. M. et al. Antidiabetic potential of novel 1, 3, 5-trisubstituted-2-thioxoimidazloidin-4-one analogues: insights into α-glucosidase, α-amylase, and antioxidant activities. *Pharmaceuticals* **15**, 1576 (2022). 36559028 10.3390/ph15121576 PMC9785777

[26] Daina, A., Michielin, O. & Zoete, V. SwissADME: a free web tool to evaluate pharmacokinetics, drug-likeness and medicinal chemistry friendliness of small molecules. *Sci. Rep.* **7**, 42717 (2017). 28256516 10.1038/srep42717 PMC5335600

[27] Keller, T. H., Pichota, A. & Yin, Z. A practical view of ‘druggability’. *Curr. Opin. Chem. Biol.* **10**, 357–361 (2006). 16814592 10.1016/j.cbpa.2006.06.014

[28] Pires, D. E., Blundell, T. L. & Ascher, D. B. PkCSM: predicting small-molecule Pharmacokinetic and toxicity properties using graph-based signatures. *J. Med. Chem.* **58**, 4066–4072 (2015). 25860834 10.1021/acs.jmedchem.5b00104 PMC4434528

[29] Ayoup, M. S. et al. Novel sulfonamide derivatives as multitarget antidiabetic agents: design, synthesis, and biological evaluation. *RSC Adv.* **14**, 7664–7675 (2024). 38440282 10.1039/d4ra01060d PMC10910856

[30] Bassin, J., Cremlyn, R. & Swinbourne, F. Chlorosulfonation of aromatic and hetero-aromatic systems. *Phosphorus Sulfur Silicon Relat. Elem.* **56**, 245–275 (1991).

[31] Alavi, S., Mosslemin, M. H., Mohebat, R. & Massah, A. R. Green synthesis of novel Quinoxaline sulfonamides with antibacterial activity. *Res. Chem. Intermed.* **43**, 4549–4559 (2017).

[32] Ugwu, D. I., Okoro, U. C., Ukoha, P. O., Gupta, A. & Okafor, S. N. Novel anti-inflammatory and analgesic agents: synthesis, molecular Docking and in vivo studies. *J. Enzyme Inhib. Med. Chem.* **33**, 405–415 (2018). 29372659 10.1080/14756366.2018.1426573 PMC7011796

[33] Ahmad, R. et al. Chiral Aryl sulfonyl hydantoins as hypoglycemic agents. *Z. Für Naturforschung B*. **55**, 203–207 (2000).

[34] Valko, M. et al. Free radicals and antioxidants in normal physiological functions and human disease. *Int. J. Biochem. Cell Biol.* **39**, 44–84 (2007). 16978905 10.1016/j.biocel.2006.07.001

[35] Ernawati, T., Mun’im, A., Hanafi, M., & Yanuar, A. In silico evaluation of molecular interactions between known α-glucosidase inhibitors and homologous α-glucosidase enzymes from Saccharomyces cerevisiae, Rattus norvegicus, and GANC-human. *Thai J. Pharm. Sci.* **42**(1), 14–20 (2018)

[36] Dash, R. P., Babu, R. J. & Srinivas, N. R. *Reappraisal and Perspectives of Clinical drug–drug Interaction Potential of α-glucosidase Inhibitors Such as Acarbose, Voglibose and Miglitol in the Treatment of Type 2 Diabetes Mellitus*4889–108 (Xenobiotica, 2018). 10.1080/00498254.2016.1275063 28010166

[37] Bernfeld, P. *Methods in Enzymology. By SP Colowick and NO Kaplan* (Academic Press Inc., 1955).

[38] Mosmann, T. Rapid colorimetric assay for cellular growth and survival: application to proliferation and cytotoxicity assays. *J. Immunol. Methods*. **65**, 55–63 (1983). 6606682 10.1016/0022-1759(83)90303-4

[39] Goyal, S. N. et al. Challenges and issues with streptozotocin-induced diabetes–a clinically relevant animal model to understand the diabetes pathogenesis and evaluate therapeutics. *Chem. Biol. Interact.* **244**, 49–63 (2016). 26656244 10.1016/j.cbi.2015.11.032

[40] Pipatpiboon, N., Pintana, H., Pratchayasakul, W., Chattipakorn, N. & Chattipakorn, S. C. DPP 4-inhibitor improves neuronal insulin receptor function, brain mitochondrial function and cognitive function in rats with insulin resistance induced by high‐fat diet consumption. *Eur. J. Neurosci.* **37**, 839–849 (2013). 23240760 10.1111/ejn.12088

[41] Park, H. et al. Discovery of novel α-glucosidase inhibitors based on the virtual screening with the homology-modeled protein structure. *Bioorg. Med. Chem.* **16**, 284–292 (2008). 17920282 10.1016/j.bmc.2007.09.036

[42] Jia, L., Liu, Y., Fu, B., Tian, Y. & Meng, X. Liquidambaric acid as a non-competitive α-glucosidase inhibitor: multi-level evidence from enzyme kinetics, molecular docking, molecular dynamics simulations, and a drosophila hyperglycaemic model. *J. Enzyme Inhib. Med. Chem.* **40**, 2497486 (2025). 40302183 10.1080/14756366.2025.2497486 PMC12044908

[43] Brayer, G. D., Luo, Y. & Withers, S. G. The structure of human pancreatic α-amylase at 1.8 Å resolution and comparisons with related enzymes. *Protein Sci.* **4**, 1730–1742 (1995). 8528071 10.1002/pro.5560040908 PMC2143216

[44] Junejo, J. A., Zaman, K., Rudrapal, M., Celik, I. & Attah, E. I. Antidiabetic bioactive compounds from tetrastigma angustifolia (Roxb.) deb and oxalis debilis kunth.: validation of ethnomedicinal claim by in vitro and in Silico studies. *South. Afr. J. Bot.* **143**, 164–175 (2021).

[45] Rasouli, H., Hosseini-Ghazvini, S. M. B., Adibi, H. & Khodarahmi, R. Differential α-amylase/α-glucosidase inhibitory activities of plant-derived phenolic compounds: A virtual screening perspective for the treatment of obesity and diabetes. *Food Funct.* **8**, 1942–1954 (2017). 28470323 10.1039/c7fo00220c

[46] HalimS.A., Jabeen, S., Khan, A. & Al-Harrasi, A. Rational design of novel inhibitors of α-glucosidase: an application of quantitative structure activity relationship and structure-based virtual screening. *Pharmaceuticals* **14**, 482 (2021). 10.3390/ph14050482 PMC8158765 34069325

[47] Suzuki, M. et al. Rebamipide, a novel antiulcer agent, attenuates Helicobacter pylori induced gastric mucosal cell injury associated with neutrophil derived oxidants. *Gut* **35**, 1375–1378 (1994). 7959190 10.1136/gut.35.10.1375 PMC1375008

[48] Chaurasiya, A. & Chawla, P. A. Synthetic strategy of 2-thioxo-4-thiazolidinone with core chemistry and biological importance. *Pharmaspire* **14**, 97–103 (2022).

[49] Kerru, N., Gummidi, L., Maddila, S., Gangu, K. K. & Jonnalagadda, S. B. A review on recent advances in nitrogen-containing molecules and their biological applications, Molecules, 25 1909. (2020). 10.3390/molecules25081909 PMC7221918 32326131

[50] Singh, V., Singh, A., Singh, G., Verma, R. K. & Mall, R. Benzoxazolyl linked benzylidene based Rhodanine and analogs as novel antidiabetic agents: synthesis, molecular docking, and in vitro studies. *Med. Chem. Res.* **30**, 1905–1914 (2021).

[51] Mermer, A. The importance of Rhodanine scaffold in medicinal chemistry: A comprehensive overview. *Mini Rev. Med. Chem.* **21**, 738–789 (2021). 33334286 10.2174/1389557521666201217144954

[52] Khan, I., Zaib, S. & Ibrar, A. New frontiers in the transition-metal-free synthesis of heterocycles from alkynoates: an overview and current status. *Org. Chem. Front.* **7**, 3734–3791 (2020).

[53] Hassan, A., Said, M., Sarg, M., Al-Zahabi, H. & Hussein, E. Utility of 2-thiohydantoin derivatives in the synthesis of some condensed heterocyclic compounds with expected biological activity. *Life Sci. J.* **10**, 16–19 (2013).

