# Supplementary material for: Synthesis and evaluation of novel thiohydantoin derivatives for antidiabetic activity using in silico in vitro and in vivo methods
Source: Sci Rep. 2025 Aug 1;15:28100. doi: 10.1038/s41598-025-13538-7 (PMC12316928; doi:10.1038/s41598-025-13538-7)
Supplement: Supplementary file 1 — Supplementary Material 1 [file 41598_2025_13538_MOESM1_ESM.docx]

**SUPPLEMENTARY DATA**


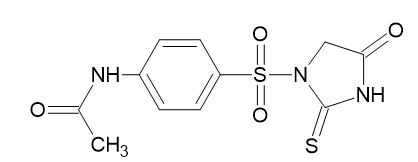

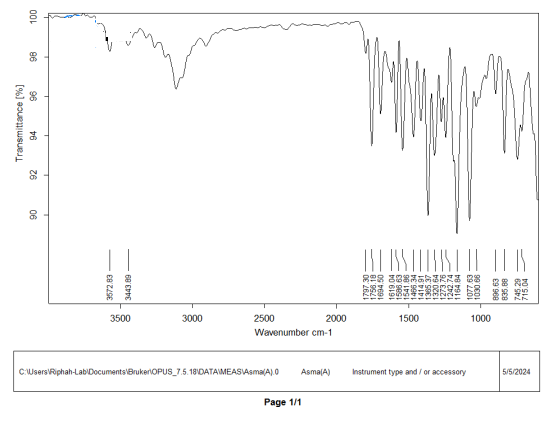


**Fig S1 : FTIR FP1**


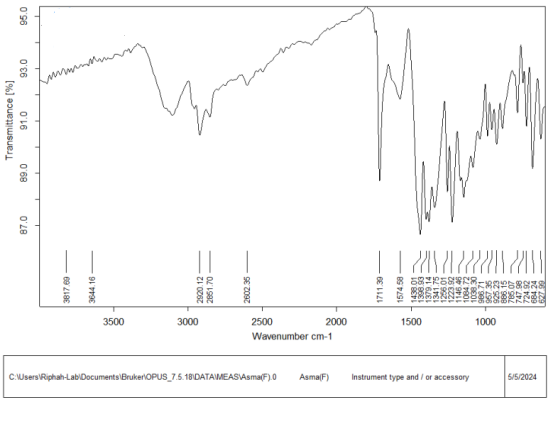


**Fig S2 : FTIR FP2**


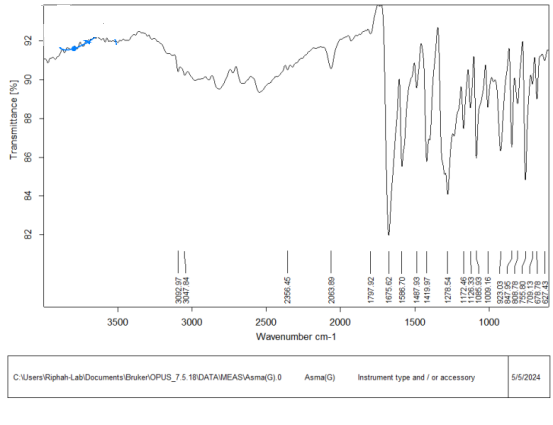


**Fig S3 : FTIR FP3**


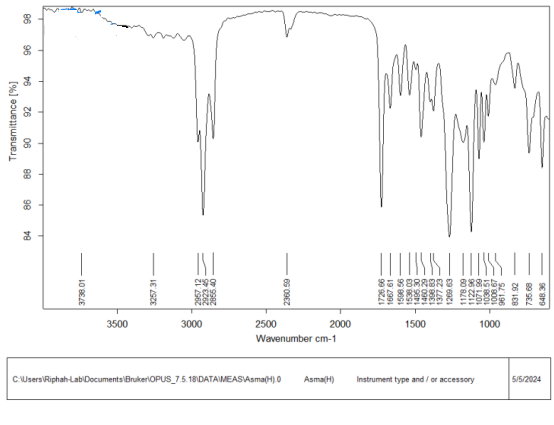


**Fig S4 : FTIR FP4**


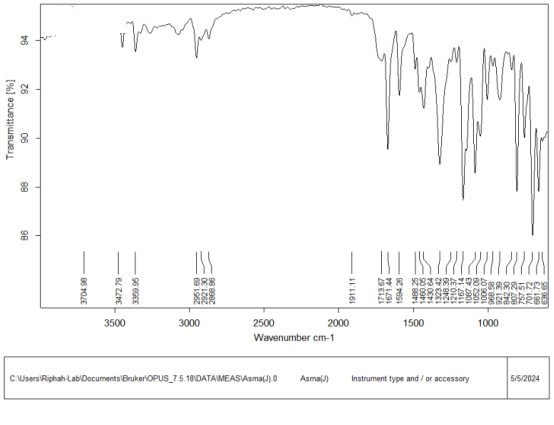


**Fig S5 : FTIR FP5**


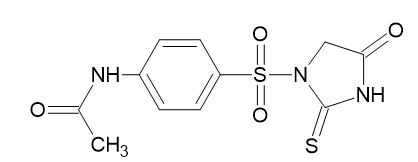

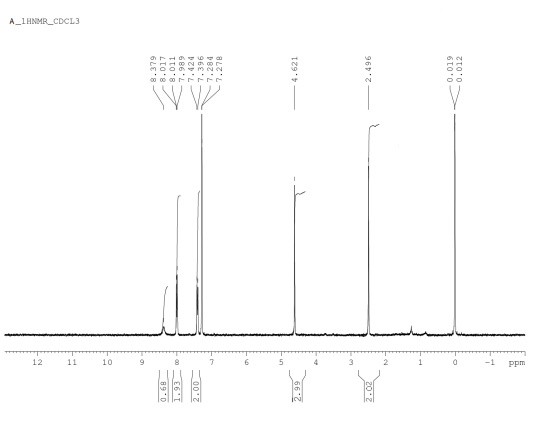


**Fig S6 : ^1^H-NMR FP1**


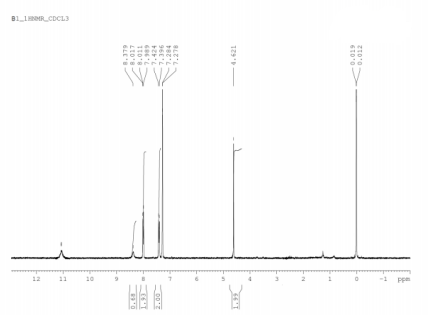

**Fig S7 : ^1^H-NMR FP2**


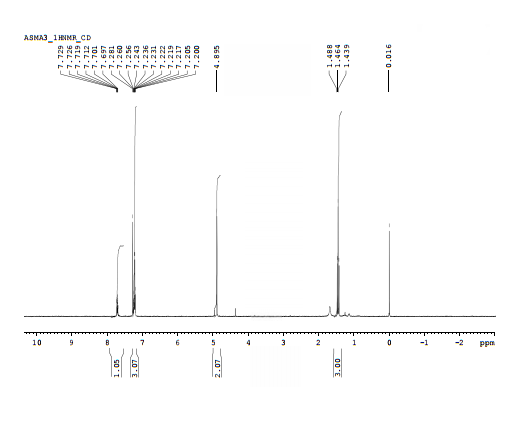

**Fig S8 : ^1^H-NMR FP3**


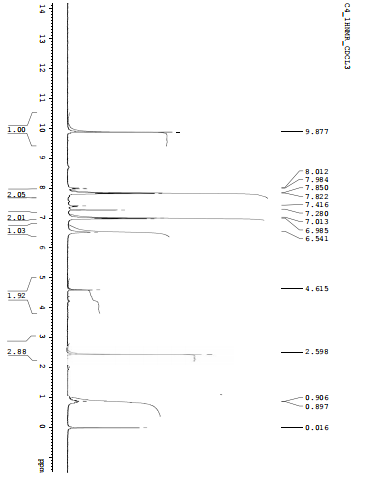

**Fig S10 : ^1^H-NMR FP4**


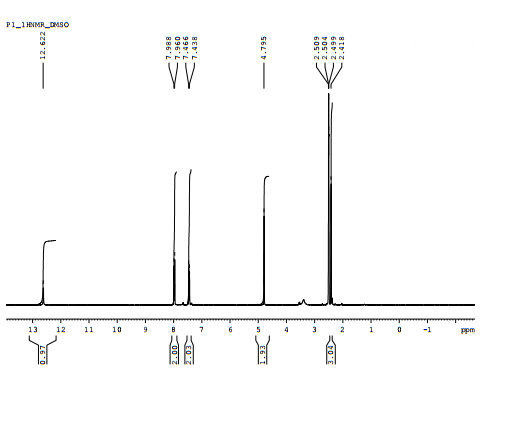

**Fig S11 : ^1^H-NMR FP5**


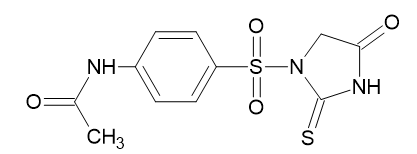

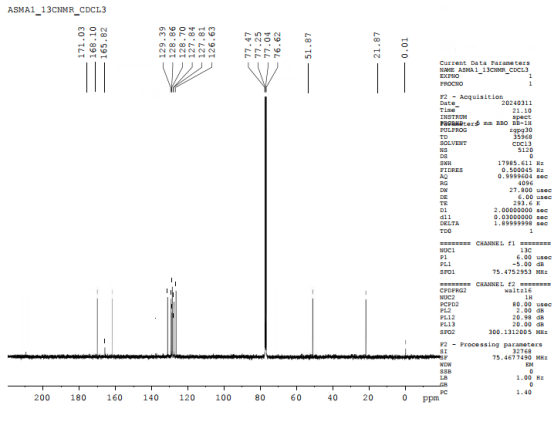


**Fig S12 : 13C-NMR FP1**


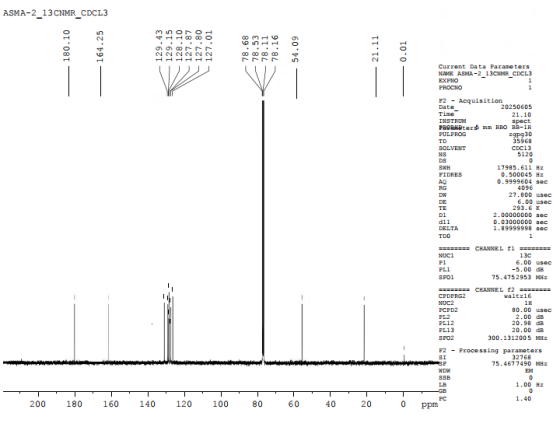

**Fig S13 : 13C-NMR FP3**


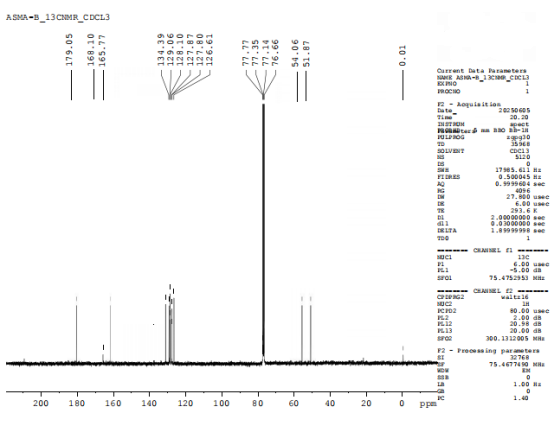

**Fig S14 : 13C-NMR FP4**


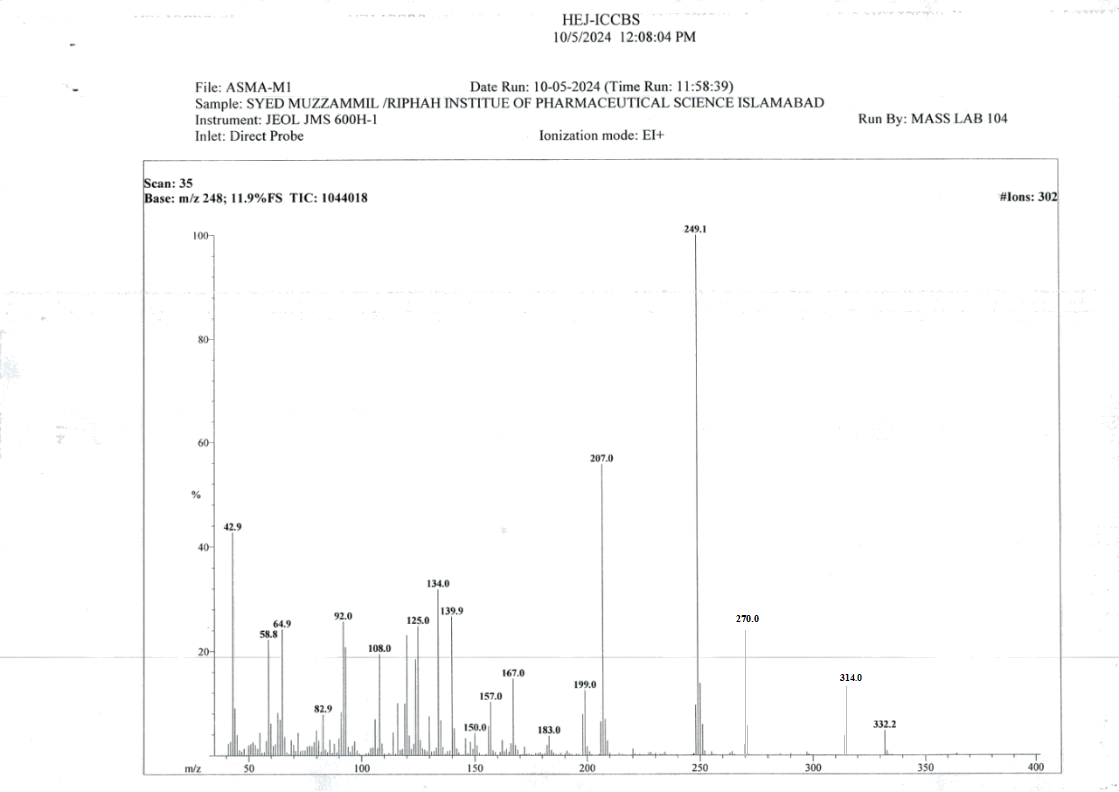

**Fig S15 : Mass Spectrum FP1**


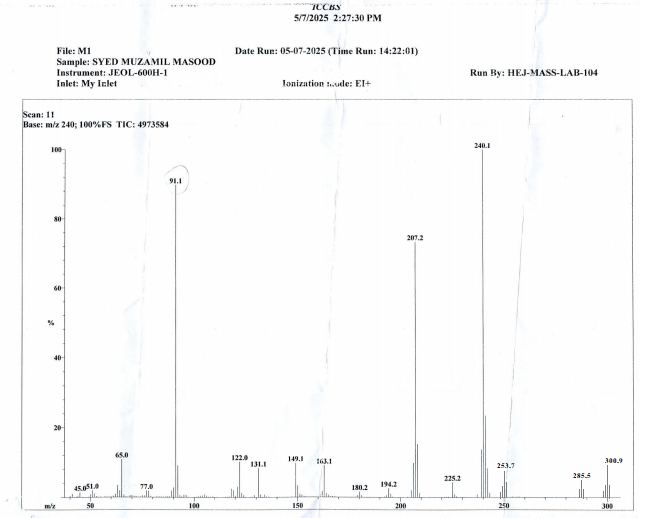

**Fig S16 : Mass Spectrum FP2**


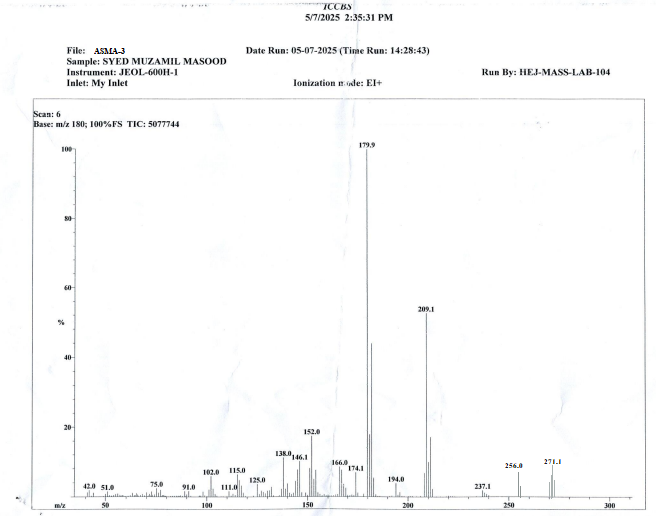

**Fig S17 : Mass Spectrum FP3**


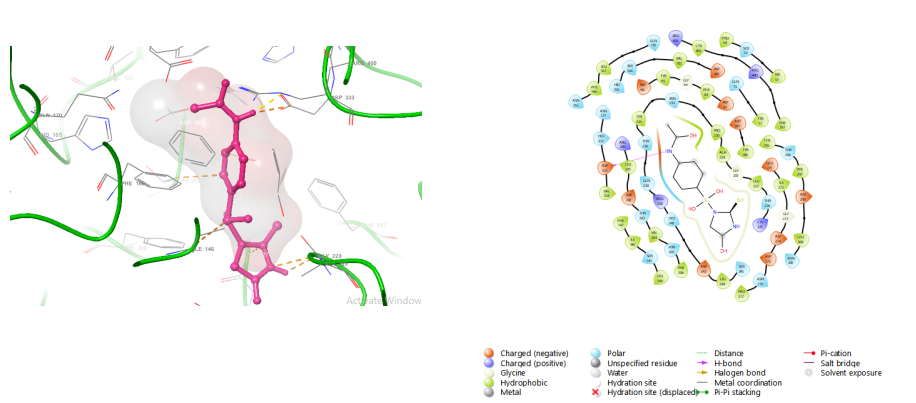


**Figure S18:** 2D and 3D poses of Molecular dockng interaction diagram FP1 against **α-glucosidase (PDB: 3wy1) .**Images were drawn using Schrödinger Release 2023-2: Maestro, Schrödinger, LLC, New York, NY, 2023.


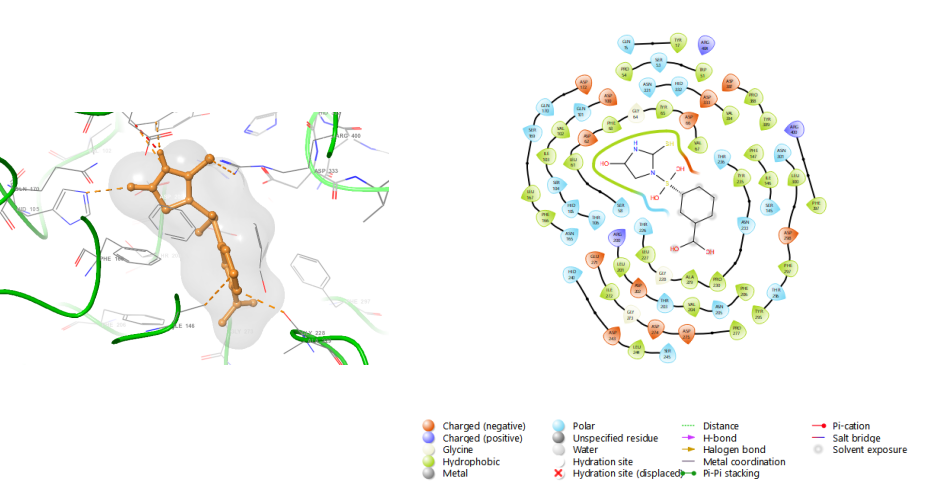


**Figure S19:** 2D and 3D poses of Molecular dockng interaction diagram FP2 against **α-glucosidase (PDB: 3wy1) .**Images were drawn using Schrödinger Release 2023-2: Maestro, Schrödinger, LLC, New York, NY, 2023.


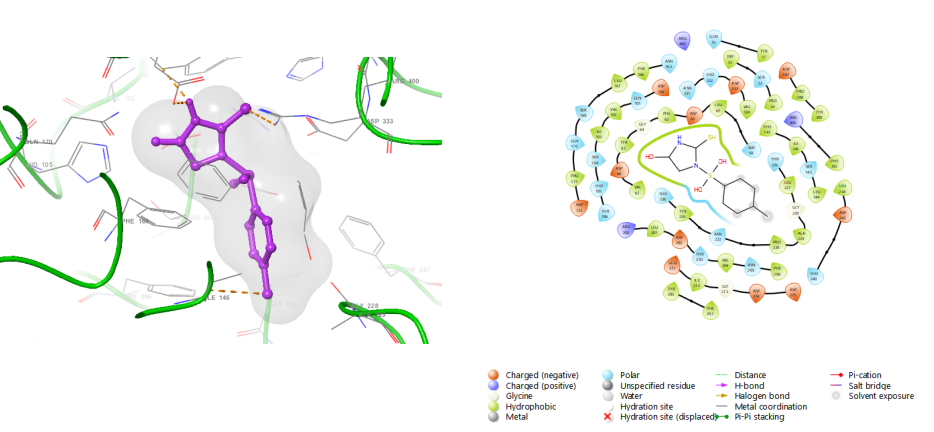


**Figure S20:** 2D and 3D poses of Molecular dockng interaction diagram FP3 against **α-glucosidase (PDB: 3wy1) .**Images were drawn using Schrödinger Release 2023-2: Maestro, Schrödinger, LLC, New York, NY, 2023.


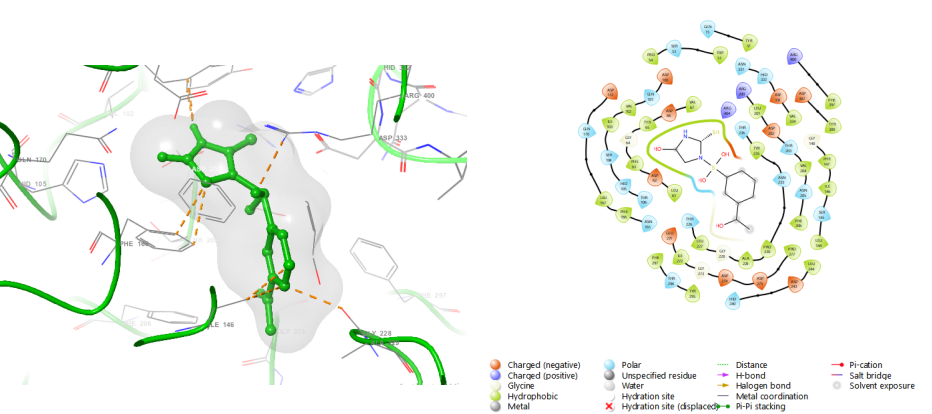


**Figure S21:** 2D and 3D poses of Molecular dockng interaction diagram FP4 against **α-glucosidase (PDB: 3wy1) .**Images were drawn using Schrödinger Release 2023-2: Maestro, Schrödinger, LLC, New York, NY, 2023.


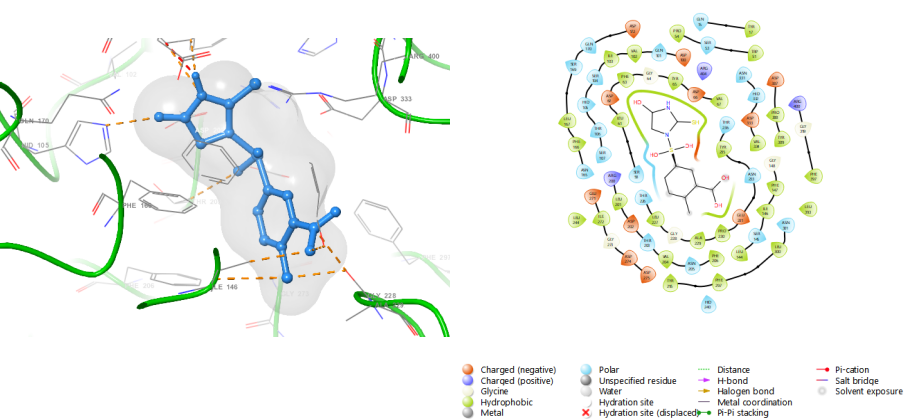


**Figure S22:** 2D and 3D poses of Molecular dockng interaction diagram FP5 against **α-glucosidase (PDB: 3wy1) .**Images were drawn using Schrödinger Release 2023-2: Maestro, Schrödinger, LLC, New York, NY, 2023.


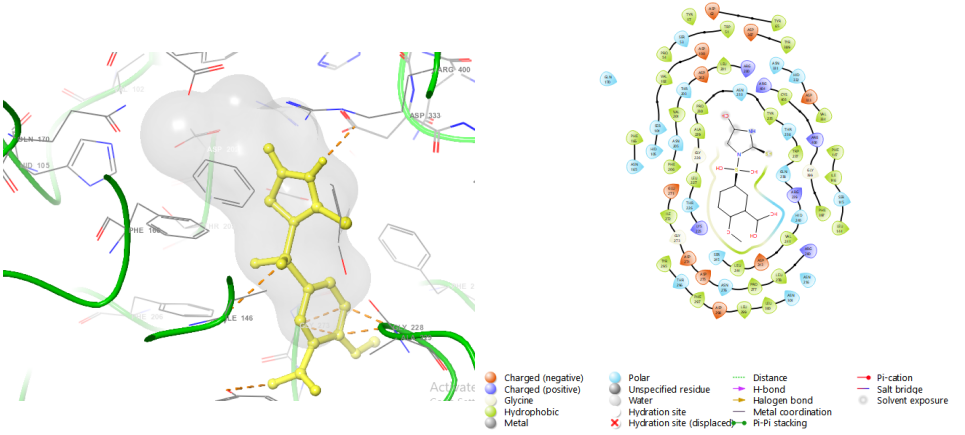


**Figure S23:** 2D and 3D poses of Molecular dockng interaction diagram FP6 against **α-glucosidase (PDB: 3wy1) .**Images were drawn using Schrödinger Release 2023-2: Maestro, Schrödinger, LLC, New York, NY, 2023.


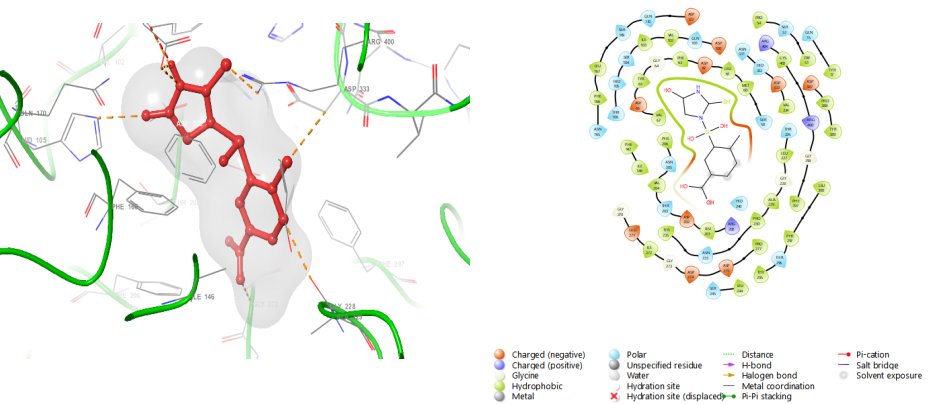


**Figure S24:** 2D and 3D poses of Molecular dockng interaction diagram FP7 against **α-glucosidase (PDB: 3wy1) .**Images were drawn using Schrödinger Release 2023-2: Maestro, Schrödinger, LLC, New York, NY, 2023.


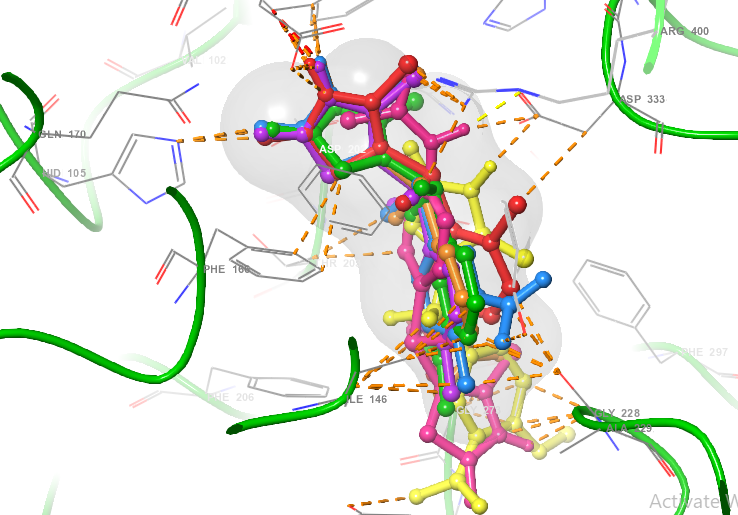


**Figure S25:** Superimpose FP1- FP7 against **α-glucosidase (PDB: 3wy1) within same binding pocket .**Images were drawn using Schrödinger Release 2023-2: Maestro, Schrödinger, LLC, New York, NY, 2023.


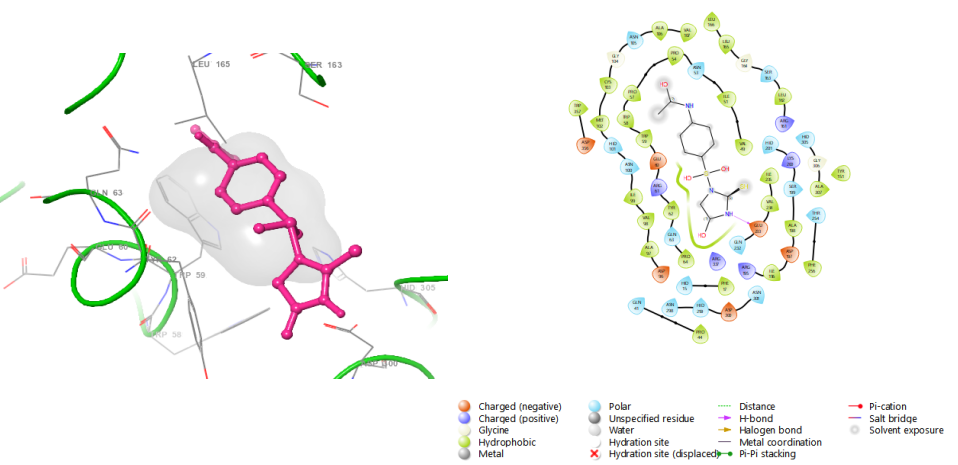


**Figure S26:** 2D and 3D poses of Molecular dockng interaction diagram FP1 against α-amylase (PDB: 3dhp). Images were drawn using Schrödinger Release 2023-2: Maestro, Schrödinger, LLC, New York, NY, 2023.


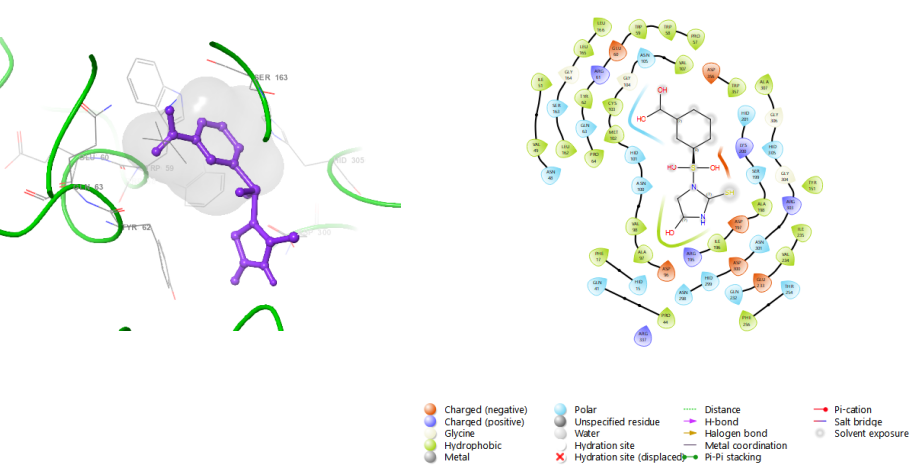


**Figure S27:** 2D and 3D poses of Molecular dockng interaction diagram FP2 against α-amylase (PDB: 3dhp) . Images were drawn using Schrödinger Release 2023-2: Maestro, Schrödinger, LLC, New York, NY, 2023.


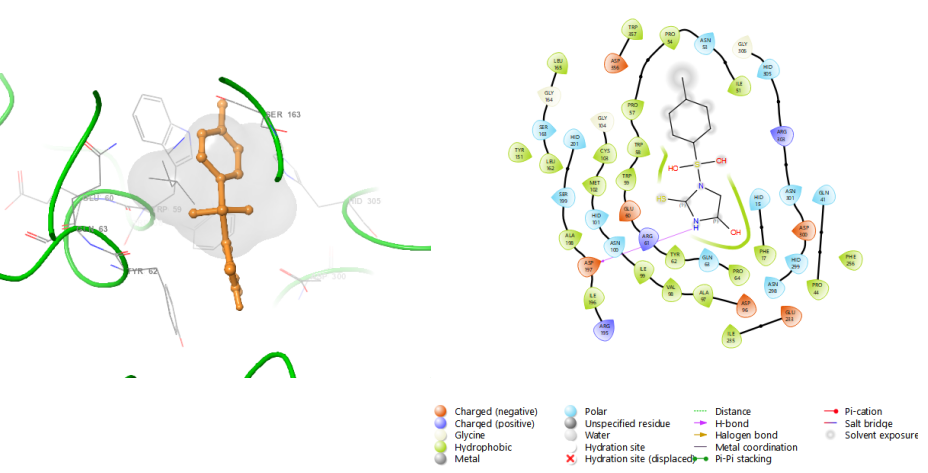


**Figure S28:** 2D and 3D poses of Molecular dockng interaction diagram FP3 against α-amylase (PDB: 3dhp) Images were drawn using Schrödinger Release 2023-2: Maestro, Schrödinger, LLC, New York, NY, 2023.


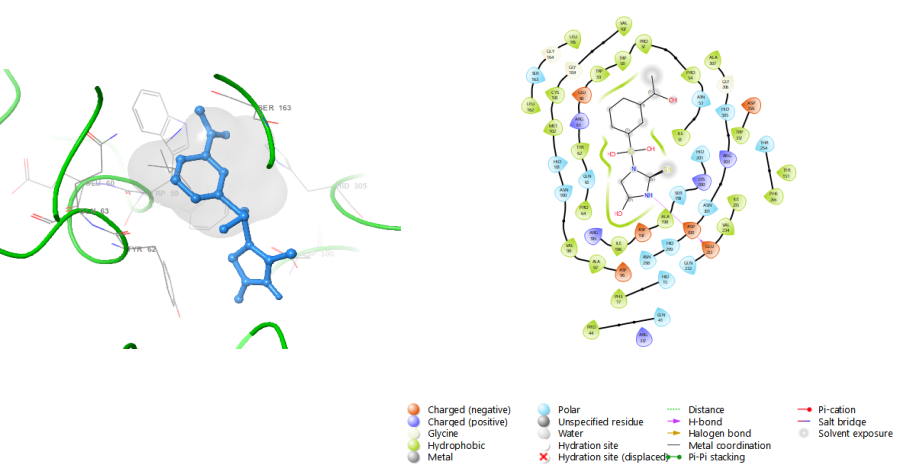


**Figure S29:** 2D and 3D poses of Molecular dockng interaction diagram FP4 against α-amylase (PDB: 3dhp) Images were drawn using Schrödinger Release 2023-2: Maestro, Schrödinger, LLC, New York, NY, 2023.


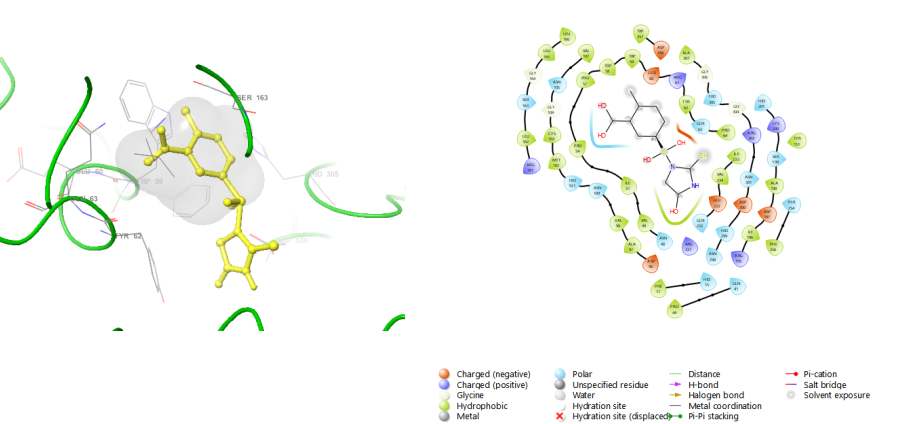


**Figure S30:** 2D and 3D poses of Molecular dockng interaction diagram FP5 against α-amylase (PDB: 3dhp)

Images were drawn using Schrödinger Release 2023-2: Maestro, Schrödinger, LLC, New York, NY, 2023.


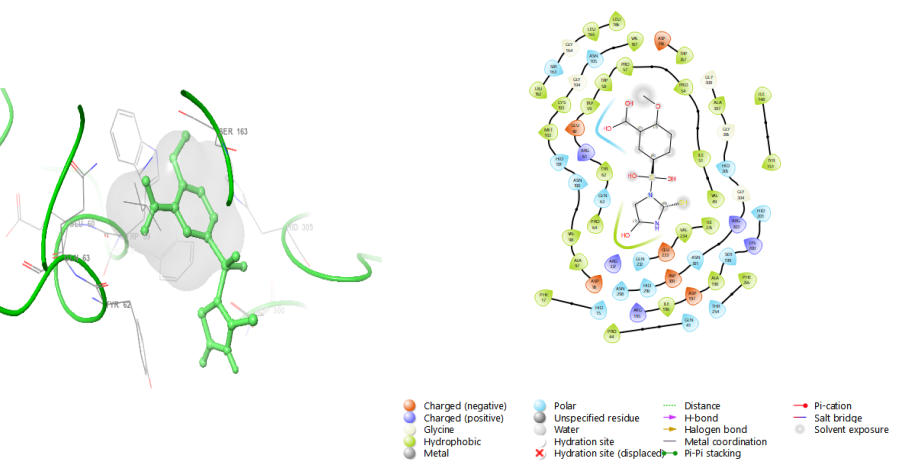


**Figure S31:** 2D and 3D poses of Molecular dockng interaction diagram FP6 against α-amylase (PDB: 3dhp)

Images were drawn using Schrödinger Release 2023-2: Maestro, Schrödinger, LLC, New York, NY, 2023.


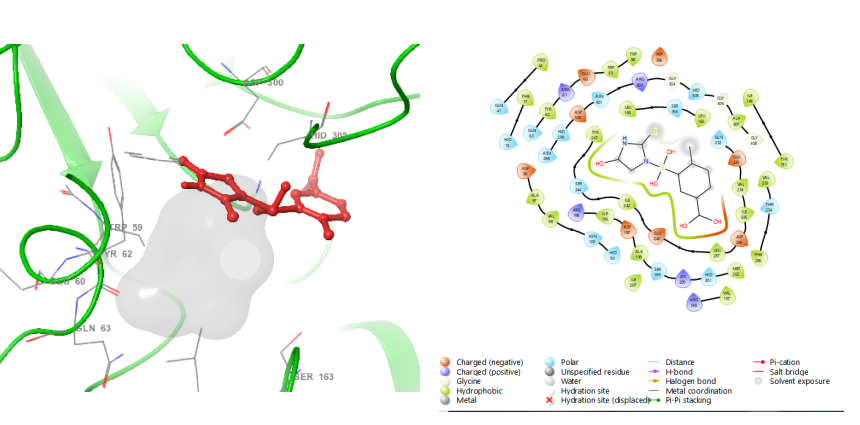


**Figure S32:** 2D and 3D poses of Molecular dockng interaction diagram FP7 against α-amylase (PDB: 3dhp)

Images were drawn using Schrödinger Release 2023-2: Maestro, Schrödinger, LLC, New York, NY, 2023.


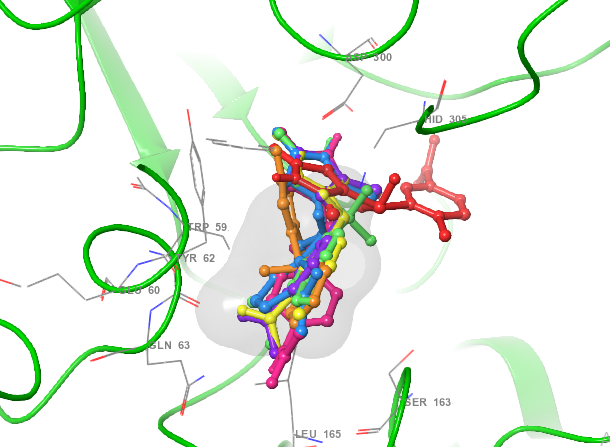
\

**Figure S33:** Superimpose FP1- FP7 against α-amylase (PDB: 3dhp) **within same binding pocket**

Images were drawn using Schrödinger Release 2023-2: Maestro, Schrödinger, LLC, New York, NY, 2023.
